# Microwave Irradiation Assists the Synthesis of a Novel Series of *bis*-Arm *s*-Triazine Oxy-Schiff Base and Oxybenzylidene Barbiturate Derivatives

**DOI:** 10.3390/molecules23112976

**Published:** 2018-11-14

**Authors:** Kholood A. Dahlous, Zainab Almarhoon, Ahmed-Yacine Badjah-Hadj-Ahmed, Zeid A. AL Othman, Ayman El-Faham

**Affiliations:** 1 Department of Chemistry, College of Science, King Saud University, P.O. Box 2455, Riyadh 11451, Saudi Arabia; kdahloos@ksu.edu.sa (K.A.D.); zalmarhoon@ksu.edu.sa (Z.A.); ybadjah@ksu.edu.sa (A.-Y.B.-H.-A.); zaothman@ksu.edu.sa (Z.A.A.O.); 2 Department of Chemistry, Faculty of Science, Alexandria University, P.O. Box 426, Ibrahimia, 12321 Alexandria, Egypt

**Keywords:** *s*-Triazine, dipodal, oxy-Schiff base, oxybenzylidene

## Abstract

A novel series of *s*-triazines incorporating 4-hydroxybenzaldehyde and 4-hydroxy-3-methoxybenzaldehyde was prepared and fully characterized. The reaction was carried out via stepwise nucleophilic aromatic substitution of chlorine atoms in cyanuric chloride. The first chlorine was substituted by different amines (morpholine, piperidine, or diethylamine) to afford 2,4-dichloro-6-substituted-1,3,5-triazine. The second and third chlorines were substituted by benzaldehyde derivatives in the presence of Na_2_CO_3_ as a HCl scavenger to afford the target products: s-triazine oxyaldehyde derivatives (dipodal). The dipodal derivatives were reacted with acid hydrazide, hydralazine, barbituric, or thiobarbituric acid derivatives using conventional heating or microwave irradiation to afford the di-arm *s*-triazine oxy-Schiff base and oxybenzylidene barbiturate derivatives in good yields. Microwave irradiation done in less solvent afforded the target product in less reaction time with good yield and purity. These types of derivatives might have special interest in coordination and medicinal chemistry.

## 1. Introduction

In the past few years, the applications of microwaves have been increasing in use and become more interesting in various fields since the first published reports on the use of microwave irradiation to carry out organic reactions by Gedye et al. in 1986 [1]. Recently, microwave-assisted organic synthesis (MAOS) has grown dramatically because it reduces reaction times and increases the yield and purity of the product by reducing the undesirable side reactions associated with conventional heating [2,3,4,5,6,7].

In addition, 1,3,5-triazine (*s*-triazine) has drawn significant interest as a perfect example for combinatorial library scaffold. The use of 1,3,5-triazine derivatives in various applications is well documented [8,9,10,11,12,13,14,15,16,17]. The most convenient method for the synthesis of 1,3,5-triazine derivatives is based on the use of the inexpensive and readily commercially available reagent 2,4,6-trichloro-1,3,5-triazine (cyanuric chloride) as the starting material. The ease of the stepwise substitution of the three chlorine atoms and the chemoselective reactivity with a variety of nucleophiles under temperature control has drawn significant interest for the synthesis of hyperbranched polymers [9,13,18,19,20], dendrimers [21], and tri-arm star-shaped molecules with applications in organic light-emitting diodes [22].

The reaction of cyanuric chloride with three equivalents of 4-hydroxybenzaldehyde that afforded the tri-oxyaldehyde (tripodal) derivatives in a single step has been reported in the literature [23,24,25,26], 2,4,6-Tris(4-formylphenoxy)-1,3,5-triazine, with three reactive peripheral aldehyde groups makes it a useful reagent for the preparation of star-shaped molecules. Gingrast et al. [23] reported the preparation of some star-shaped thiosemicarbazones containing the *s*-triazine ring as a new class of biologically active compounds. Later, tripodal was reported as a useful imprinting material on silica, which provides a site of three organized amino groups that can be further modified with various functional groups [23]. Recently, Koc et al. [25] reported the use of tri-oxyaldehyde derivatives for the preparation of several oxy-Schiff bases, which were used as tripodal-trinuclear systems formed by the 1,3,5-tricarboxylate bridge for complexation with iron (III). Later, the same author reported the synthesis, electrochemical behavior, and antimicrobial activity of four tripodal–benzimidazole derivatives based on the reaction of tris(4-oxyaldehyde)-1,3,5-triazone with different *o*-phenylene diamine derivatives [26]. Recently, Celikbilek and Koc [27] reported the dipodal oxy-Schiff base derived from the methoxy-*s*-triazine and their salen and salophen complexes.

Due to the presence of the azomethine (R-NH-N=C-R) in Schiff bases moiety, these compounds have gained great importance in medicinal chemistry as well as coordination chemistry [28,29,30,31,32,33,34]. In addition, barbiturate derivatives have shown remarkable biological activity [35,36] and are considered as important intermediates for the synthesis of several heterocyclic compounds [37,38]. Herein, we report the synthesis and characterization of a novel series of *bis*-arm *s*-triazine oxy-Schiff bases and oxybenzylidene barbiturate derivatives using conventional heating and microwave irradiation as a new template, which might be important in coordination and medicinal chemistry for researchers in this field.

## 2. Results and Discussion

**Scheme 1:**
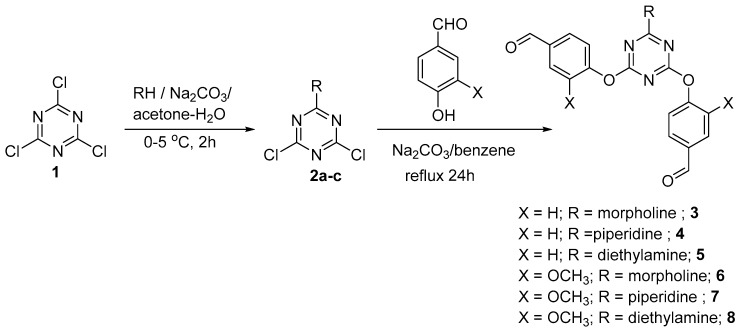
Synthetic pathway for dipodal of *s*-triazine derivatives.

Due to the different reactivities of the three chlorine atoms in cyanuric chloride, these chlorine atoms could be replaced using different nucleophiles by controlling the temperature, i.e., temperature-controlled selectivity. Moreover, cyanuric chloride also shows good selectivity toward nucleophilic substitution by the amino-to-hydroxyl group, which is called functional group selectivity [39,40]. In the present work, we took this advantage for the preparation of di-arm derivatives. First, cyanuric chloride **1** was reacted with different amines—morpholine, piperidine, and diethylamine—to afford 2,4-dichloro-6-substituted-*s*-triazine **2a**–**c**. In the second step, compounds **2a**–**c** were reacted with two equiv. of 4-hydroxybenzaldehyde derivatives to afford dipodal derivatives **3**–**8** with high yield and purity as observed from their spectral data (Scheme 1).

**Scheme 2:**
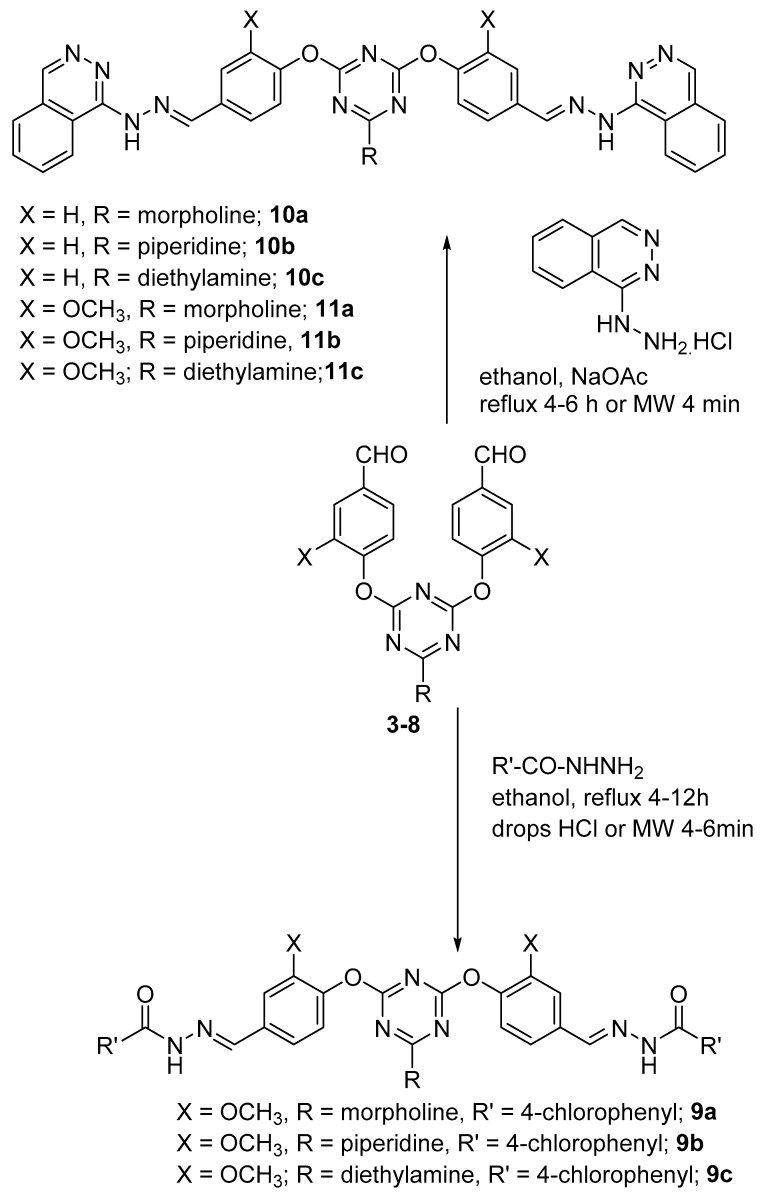
Synthesis of dipodal oxy-Schiff base *s*-triazine derivatives.

For preparation of di-arm derivatives **9**–**11**, compounds **3**–**8** were reacted with acid hydrazide or hydralazine in ethanol as a solvent to afford the target products **9a**–**c**, **10a**–**c**, and **11a**–**c** with good yields (Scheme 2). The reaction was repeated using microwave irradiation (60 °C, 600 W, 4–6 min for the hydrazide reaction; 4 min for the hydralazine reaction). Microwave irradiation afforded the products in shorter reaction times with higher yields and purities, as observed from their spectral data and UPLC-MS data (see experimental section).

The Infrared (IR) spectra for the hydralazine series **10a**–**c** and **11a**–**c** were verified by the appearance of very characteristic ν (C=N_T_) vibrations in the region 1531 cm^–1^. The amide ν (N-H) and ν (C=O) bands were observed in the regions 3210 cm^–1^ and 1669 cm^–1^, respectively, and these triazine derivatives showed another important infrared band in the region 1384 cm^−1^ that was attributed to C_T-O-Ar_ stretching. The -C=N-_imine_ stretching vibrations that were observed in the region 1594 cm^–1^ bands are the distinguishing feature of triazine.

**Figure 1:**
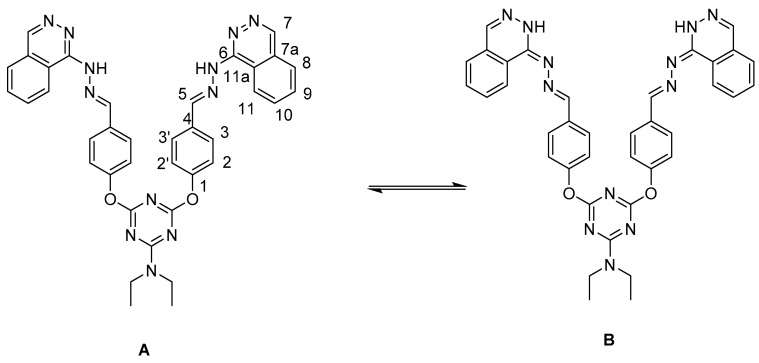
Structure of compound **11c**.

The ^1^H-NMR spectrum of **11c** (Figure 1) as a prototype showed triplet and multiplet peaks at δ 0.97 and δ 3.32 ppm related to the ethyl residue (CH_3_ and CH_2_, respectively), a doublet peak at δ 7.28 ppm for 4H related to the H_3,3’_, and a multiplet peak in the range δ 7.72–7.77 ppm for 6H corresponding to the aromatic protons H_9,10,11_. In addition, two doublets appeared at δ 8.09 representing 4H_2,2’_ and 2H_8_, and the peak at δ 8.28 ppm represented H_7_. The proton related to the HC=N appeared as a singlet at δ 8.46 ppm. Due to the possibility of the tautomeric structure of compound **11c**, as indicated in Figure 1, the NH appeared at δ 12.15 ppm, which indicated the high acidic character and agreed with the proposed structure **B** more than **A**, and this observation agreed with the recently reported data by our group [41]. The ^13^C-NMR spectrum of **11c** exhibited two peaks for the diethylamino residue at δ 12.6 and 41.5 ppm related to CH_3_ and CH_2_, respectively, and absorption peaks at δ 165.8 and 171.5 ppm related to (2-C=N-_O–Ar_) and (C=N-diethyl amino), respectively, besides peaks at δ 121.8 (C_11a_), 123.6 (C_11_), 126.1 (C_2_,_2’_), 126.4 (C_7a_), 127.0 (C_8_), 128.9 (C_3,3’_), 131.7 (C_1_), 132.9 (C_10_), 137.6 (C_9_), 148.5 (C_7_), 152.3 (C_5_), and 152.9 (C_1_) ppm.

The UPLC-MS *m*/*z* for compound **11c** showed the exact molar mass (found *m*/*z*: 677.5 [M + 1]) calculated for C_37_H_32_N_12_O_2_ (676.75) using direct infusion to the UPLC-MS instrument with electrospray positive ionization (see Supplementary Materials).

**Scheme 3:**
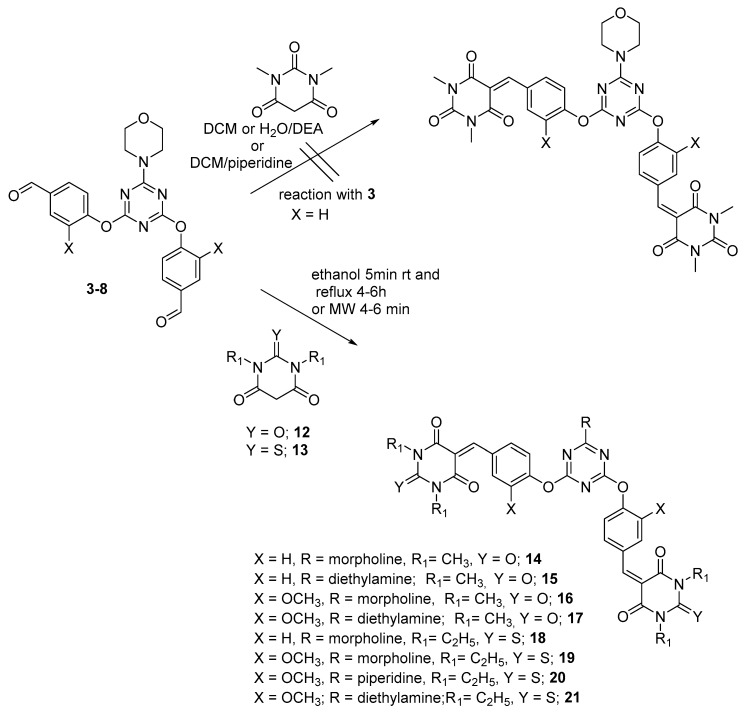
Synthesis of dipodal oxybenzylidene barbiturate derivatives.

The results obtained from the synthesis of oxy-Schiff base derivatives encouraged us to try the reaction of the dipodal with barbituric acid derivative **12** (1,3-dimethylpyrimidine-2,4,6(*1H,3H,5H*)-trione) under mild conditions (Scheme 3).

As a first attempt, we tried the reaction of dipodal **3** with barbituric acid derivatives **12** using ethanol as a solvent in the presence of piperidine as a catalyst [42]. From the thin layer chromatography (TLC) observation, the starting material had almost completely disappeared after 2–3 h at room temperature, while three more spots were observed. After the workup and separation of the crude product, the NMR spectrum showed more than one product that failed to be isolated or purified.

As a second attempt, we tried other conditions using dichloromethane (DCM) or water as a solvent in the presence of diethylamine as a catalyst [43,44]; the same problem was observed, where more than one product was formed. Finally, we followed the method described by Jursic [45], in which ethanol was used without any catalyst. Better results were obtained, but the starting material was observed even after stirring at room temperature for 24 h or refluxing for 6 h, as observed from following the reaction by TLC (ethylacetate-hexane, 4:6). Using microwave irradiation improved the yield of the product and the reaction was completed in 4 min, where TLC observed none of the starting material.

Accordingly, for the reaction using dipodal **6** with compound **12** in ethanol and refluxed for 4–6 h (Scheme 3), complete conversion was observed after 4 h, as observed by TLC (ethylacetate-hexane; 4:6). The solvent was removed under reduced pressure and the crude yellow product was recrystallized from CHCl_3_–hexane (1:1) to afford pure product **14**.

By repeating the reaction using microwave irradiation in ethanol as a solvent, the reaction afforded the target product in 4 min with higher yield and purity (see experimental section).

## 3. Materials and Methods

### 3.1. General

Cyanuric chloride, 4-hydroxybenzaldehyde, and 4-hydroxy-3-methoxybenzaldehyde were purchased from Sigma-Aldrich (Chemie GmbH, Taufkirchen, Germany). Melting points were recorded in open capillary tubes and were uncorrected (Sigma–Aldrich Chemie GmbH, Taufkirchen, Germany). IR spectra (KBr in cm^–1^) were recorded on a Shimadzu 8201 PC FTIR spectrophotometer (Shimadzu, Ltd., Tokyo, Japan). ^1^H and ^13^C-NMR spectra were recorded using on a JEOL-NMR spectrometer (JEOL, Ltd., Tokyo, Japan) (400, 850 MHz), and the chemical shifts were reported in δ ppm. The purity of the compounds was monitored by TLC silica (Type 60 GF254, Merck) and visualized by UV light at 254 nm. Elemental analyses were performed on a Perkin-Elmer 2400 elemental analyzer (PerkinElmer, Inc., Waltham, MA, USA), and the values found were within ±0.3% of the theoretical values. Microwave irradiation was performed in a multimode reactor with a 1400 W maximum magnetron (Synthos 3000, Aton Paar GmbH, Ostfildern, Germany). UPLC-MS conditions were as follows: The instruments used were a Waters Acquity UPLC system (Waters Corp., Milford, MA, USA) and a triple quadrupole (TQD) mass spectrometer equipped with a Z-electrospray interface. The parameters of the electrospray ionization source were as follows: capillary voltage 3.0 kV; cone voltage 28 V; desolvation gas was nitrogen with a flow of 800 L/h; cone gas was nitrogen with a flow of 70 L/h; source temperature 120 °C; and desolvation temperature 300 °C. Analysis was done in full scan mode with positive ionization in the mass range 50–850 Da. Data acquisition and processing were done using Waters MassLynx software.

### 3.2. General Method for Synthesis of Dipodal ***3***–***8***

The synthesis of dipodal **3**–**8** was performed in two steps. First, cyanuric chloride was reacted with different amines—morpholine, piperidine, benzylamine, or diethylamine—following the reported methods [41] to afford 2,4-dichloro-6-substituted *s*-triazine **2a**–**c** with good yields and purity. The spectral data were in good agreement with the reported data [41].

Second, compounds **2a**–**c** were reacted with the 4-hydroxybenzaldehyde derivatives as follows: 2,4-Dichloro-6-substituted-1,3,5-triazine **2a**–**c** (10 mmol) were added portionwise to a suspended solution of 4-hydroxybenzaldehyde or 4-hydroxy-3-methoxybenzaldehyde (22 mmol) and Na_2_CO_3_ (50 mmol) in 100 mL of benzene at room temperature. The reaction mixture was refluxed for 24 h and then cooled to room temperature. Water (100 mL) was added, and the mixture was extracted with ethylacetate (2 × 100 mL). The organic layer was collected and washed twice with a 10% solution of Na_2_CO_3_, dried over MgSO_4_, and filtered, and then the solvent was removed under vacuum to afford an off-white solid, which crystallized from ethylacetate–hexane to afford the pure products **3**–**8** at a yield of 80–88%.

*4,4′-((6-Morpholino-1,3,5-triazine-2,4-diyl)bis(oxy))dibenzaldehyde***3**. White powder in yield 88%; mp 157–158 °C; ^1^H-NMR (CDCl_3_): δ 3.63 (brs, 8H, 4CH_2_), 7.29 (d, 4H, *J* = 8.4 Hz), 7.88 (dd, 4H, *J* = 8.0 Hz, *J* = 2.0 Hz), 9.96 (s, 2H, H_ald_) ppm; ^13^C-NMR (CDCl_3_): δ 44.0, 66.3, 122.4, 131.1, 133.8, 156.5, 166.5, 171.7, 190.8 ppm. Anal. Calcd for C_21_H_18_N_4_O_5_ (406.40): C, 62.07; H, 4.46; N, 13.79; found: C, 62.26; H, 4.59; N, 13.91.

*4,4′-((6-(Piperidin-1-yl)-1,3,5-triazine-2,4-diyl)bis(oxy))dibenzaldehyde***4** [46]. White powder in yield 86%; mp 125–127 °C. ^1^H-NMR (CDCl_3_): δ 1.49 (brs, 4H, 2CH_2_), 1.59 (brs, 2H, CH_2_), 3.59 (t, 4H, *J* = 6.0 Hz, 2N-CH_2_), 7.29 (d, 4H, *J* = 8.8 Hz), 7.88 (d, 4H, *J* = 8.8Hz), 9.95 (s, 2H, H_ald_) ppm; ^13^C-NMR (CDCl_3_): δ 24.1, 25.3, 44.6, 122.2, 130.8, 133.4, 156.5, 165.7, 171.4, 190.7 ppm. Anal. Calcd for C_22_H_20_N_4_O_4_ (404.43): C, 65.34; H, 4.98; N, 13.85; found: C, 65.54; H, 5.04; N, 14.09.

*4,4′-((6-(Diethylamino)-1,3,5-triazine-2,4-diyl)bis(oxy))dibenzaldehyde***5** [47]. White powder in yield 86%; mp 127–129 °C. ^1^H-NMR (CDCl_3_): δ 0.97 (t, 6H, *J* = 6.4 Hz, 2CH_3_), 3.32 (q, 4H, *J* = 9.6 Hz, *J* = 7.6 Hz, 2CH_2_), 7.26 (d, 4H, *J* = 8.8 Hz), 7.81 (d, 4H, *J* = 8.8 Hz), 9.89 (s, 2H, H_ald_) ppm; ^13^C-NMR (CDCl_3_): δ 13.2, 42.6, 122.9, 131.4, 134.1, 157.2, 166.4, 171.9, 191.4, 191.4 ppm. Anal. Calcd for C_21_H_20_N_4_O_4_ (392.42): C; 64.28; H, 5.14; N, 14.28; found: C, 64.41; H, 5.28; N, 14.51.

*4,4′-((6-Morpholino-1,3,5-triazine-2,4-diyl)bis(oxy))bis(3-methoxybenzaldehyde)***6**. White powder in yield 81%; mp 192–195 °C. ^1^H-NMR (CDCl_3_): δ 3.62 (s, 8H, CH_2_), 3.81 (s, 6H, 2OCH_3_), 7.24 (d, 2H, *J* = 8.8 Hz), 7.41(d, 4H, *J*= 8.0 Hz, Ar-H), 9.9 (s, 2H, H_ald_) ppm; ^13^C-NMR (CDCl_3_): δ 43.9, 55.9, 110.7, 123.1, 124.6, 134.9, 146.0, 152.1, 166.8, 171.7, 190.9 ppm. Anal. Calcd for C_23_H_22_N_4_O_7_: C, 59.22; H, 4.75; N, 12.01; found: C, 59.45; H, 4.92; N, 12.28.

*4,4′-((6-(Piperidin-1-yl)-1,3,5-triazine-2,4-diyl)bis(oxy))bis(3-methoxybenzaldehyde)***7** [47]. White powder in yield 90%; mp 152–153 °C. ^1^H-NMR (CDCl_3_): δ 1.49 (brs, 4H, 2CH_2_), 1.61 (brs, 2H, CH_2_), 3.58 (t, 4H, *J* = 5.2 Hz, 2CH_2_), 3.82 (s, 6H, 2O-CH_3_), 7.24–7.27 (m, 2H, Ar-H), 7.41–7.43 (m, 4H, Ar-H), 9.92 (s, 2H, H_ald_) ppm; ^13^C-NMR (CDCl_3_): δ 24.4, 25.6, 44.8, 56.0, 110.7, 123.2, 124.7, 134.8, 146.3, 152.2, 166.2, 171.6, 191.1 ppm. Anal. Calcd for C_24_H_24_N_4_O_6_ (464.48): C, 62.06; H, 5.21; N, 12.06; found: C, 62.28; H, 5.39; N, 12.32.

*4,4′-((6-(Diethylamino)-1,3,5-triazine-2,4-diyl)bis(oxy))bis(3-methoxybenzaldehyde)***8** [48]. White powder in yield 91%; mp 118–119 °C. ^1^H-NMR (CDCl_3_): δ 0.96 (t, 6H, *J* = 7.6, 2CH_3_), 3.32 (q, 4H, *J* = 6.8 Hz, *J* = 7.2 Hz, 2CH_2_), 3.82 (s, 6H, 2O-CH_3_), 7.26 (d, 2H, *J* = 8.0 Hz, Ar-H), 7.41–7.42 (m, 4H, Ar-H), 9.91 (s, 2H, H_ald_); ^13^C-NMR (CDCl_3_): δ 12.7, 42.1, 56.0, 110.5, 123.3, 124.7, 134.8, 146.3, 152.3, 171.4, 191.1 ppm; Anal. Calcd for C_23_H_24_N_4_O_6_ (452.47): C, 61.05; H, 5.35; N, 12.38; found: C, 61.29; H, 5.47; N, 12.63.

### 3.3. General Procedure for Preparation of Bis-Arm s-Triazine oxy-Schiff Base Derivatives ***9a***–***c***, ***10a***–***c***, and ***11a***–***c***

**Method A:** Conventional heating

A suspension of the dipodal derivatives **3**–**8** (10 mmol) in 30 mL of absolute ethanol was added to a solution of 4-chlorobenzohydrazide (20 mmol) or hydralazine hydrochloride (20 mmol) in 10 mL of ethanol containing 1–3 drops of concentrated HCl (for the reaction with hydralazine, an equivalent amount of NaOAc was used). The reaction mixture was refluxed for 4–12 h (with hydrazide) or 4–6 h (with hydralazine) until the reaction was completed (TLC:ethyl acetate–hexane, 6:4; CHCl_3_:EtOH, 8:2). After cooling, the resulting solid was filtered and washed with ethanol, dried, and then recrystallized from ethylacetate.

**Method B:** Microwave irradiation

A mixture of the dipodal derivatives **3**–**8** (10 mmol), 4-chlorobenzohydrazide (20 mmol), or hydralazine hydrochloride (20 mmol) in 10 mL of ethanol containing 1 drop of concentrated HCl or sodium acetate (20 mmol) in the case of hydralazine were mixed at room temperature. The reaction mixture was then irradiated in a microwave using a multimode reactor (Synthos 3000, Aton Paar GmbH) at 60 °C and 600 W for 4–6 min (6 min for 4-chlorobenzohydrazide and 4 min for hydralazine). After cooling, the resulting solid was washed with ethanol, dried, and then recrystallized from ethylacetate.

*N′,N′′′-((1Z,1′Z)-(((6-Morpholino-1,3,5-triazine-2,4-diyl)bis(oxy))bis(3-methoxy-4,1-phenylene))bis(methanylylidene))bis(4-chlorobenzohydrazide)***9a**. Yellow powder in yield 67% (A; reaction time 8 h), 89% (B, 6 min); mp 279–280 °C. IR (KBr): 3192 (NH), 1650 (C=O), 1594, 1550 (C=N) cm^−1^. ^1^H-NMR (DMSO-*d*_6_): δ 3.53 (s, 4H, 2CH_2_), 3.56 (s, 4H, 2CH_2_), 3.81 (s, 6H, 2O-CH_3_), 7.25 (d, 2H, *J* = 7.7 Hz), 7.48 (dd, 2H, *J* = 7.65 Hz, *J* = 0.9 Hz), 7.48 (s, 2H, Ar-H), 7.28 (d, 4H, Ar-H), 7.93 (d, 4H, Ar-H), 7.94 (s, 2H_ald_), 11.95 (s, 2H) ppm; ^13^C-NMR (DMSO-*d*_6_): δ 43.6, 55.9, 65.5, 109.9, 120.7, 123.0, 128.6, 129.4, 132.9, 135.3, 136.9, 141.9, 147.6, 151.3, 162.2, 171.5 ppm. Anal. Calcd for C_37_H_32_Cl_2_N_8_O_7_ (771.61): C, 57.59; H, 4.18; N, 14.52; found: C, 57.84; H, 4.33; N, 14.79. UPLC-MS: calc. m/z: 771.61 (M); found: 773.37 (M + 2), 775.39 (M + 4).

*N′,N′′′-((1Z,1′Z)-(((6-(Piperidin-1-yl)-1,3,5-triazine-2,4-diyl)bis(oxy))bis(3-methoxy-4,1-phenylene))bis(methanylylidene))bis(4-chlorobenzohydrazide)***9b**. White powder in yield 76% (A, reaction time 12 h), 89% (B, 6 min); mp 280–283 °C. IR (KBr): 3202 (NH), 1654 (C=O), 1594, 1531 (C=N) cm^−1^. ^1^H-NMR (DMSO-*d*_6_): δ 1.43 (s, 4H, 2CH_2_), 1.56 (s, 2H, CH_2_), 3.41 (s, 4H, 2CH_2_), 3.82 (s, 6H, 2O-CH_3_), 7.25 (d, 2H, *J* = 7.7 Hz), 7.30 (dd, 2H, *J* = 7.7 Hz), 7.48 (s, 2H), 7.61 (d, 4H, *J* = 8.5 Hz), 7.93 (d, 4H, *J* = 7.65 Hz), 8.45 (s, 2H, NH), 11.95 (s, 2H) ppm; ^13^C-NMR (DMSO-*d*_6_): δ 23.8, 25.1, 44.0, 55.9, 109.8, 120.7, 123.0, 132.0, 132.8, 136.6, 142.1, 147.7, 151.4, 162.2, 165.6, 171.4 ppm. Anal. Calcd for C_38_H_34_Cl_2_N_8_O_6_ (769.76): C, 59.30; H, 4.45; N, 14.56; found: C, 59.53; H, 4.61; N, 14.78. UPLC-MS: calc. m/z: 769.76 (M); found 771.49 (M + 2).

*N′,N′′′-((1Z,1′Z)-(((6-(Diethylamino)-1,3,5-triazine-2,4-diyl)bis(oxy))bis(3-methoxy-4,1-phenylene))bis(methanylylidene))bis(4-chlorobenzo hydrazide)***9c**. White powder in yield 78% (A, reaction time 12 h), 87% (B, 6 min), mp 276–279 °C. IR (KBr): 3210 (NH), 1696 (C=O), 1594, 1531 (C=N), cm^−1^. ^1^H-NMR (DMSO-*d*_6_): δ 1.05 (s, 6H, 2CH_3_), 3.40 (s, 4H, 2CH_2_), 3.82 (s, 6H, CH_3_), 7.27 (d, 2H, *J* = 7.65 Hz), 7.31 (d, 2H, *J* = 6.8 Hz), 7.49 (s, 2H), 7.60 (s, 4H), 7.94 (d, 4H, *J* = 8.5 Hz), 8.45 (s, 2H, NH), 11.69 (s, 2H) ppm; ^13^C-NMR (DMSO-*d*_6_): δ 12.6, 41.6, 55.8, 109.7, 120.7, 123.1, 128.6, 129.6, 132.8, 136.8, 142.1, 147.7, 151.5, 162.2, 165.7, 171.4 ppm. Anal. Calcd for C_37_H_34_Cl_2_N_8_O_6_ (757.63): C, 58.66; H, 4.52; N, 14.79; found: C, 58.81; H, 4.69; N, 14.99. UPLC-MS: calc. m/z: 757.63 (M); found 758 (M + 1).

*4-(4,6-bis(4-((E)-(2-(Phthalazin-1-yl)hydrazono)methyl)phenoxy)-1,3,5-triazin-2-yl)morpholine***10a**. Yellow powder in yield 78% (A, reaction time 4 h), 91% (B, 4 min); mp 238–241 °C. IR (KBr): 3398 (NH), 1582, 1528 (C=N) cm^−1^. ^1^H-NMR (DMSO-*d*_6_): δ 3.57 (brs, 8H, 4CH_2_), 7.27 (d, 4H, *J* = 8.8 Hz), 7.68–7.76 (m, 6H), 8.10 (m, 6H, *J* = 8.4 Hz), 8.26 (d, 2H, *J* = 7.2 Hz), 8.45 (s, 2H), 12.15 (s, 2H, NH) ppm; ^13^C-NMR (DMSO-*d*_6_): δ 43.6, 65.5, 121.7, 123.6, 126.1, 126.4, 127.0, 129.1, 131.7, 132.3, 132.9, 137.6, 148.5, 152.2, 152.7, 166.2, 171.7 ppm. Anal. Calcd for C_37_H_30_N_12_O_3_ (690.26): C, 64.34; H, 4.38; N, 24.33; found: C, 64.56; H, 4.55; N, 24.61.

*1,1′-(((1E,1′E)-(((6-(Piperidin-1-yl)-1,3,5-triazine-2,4-diyl)bis(oxy))bis(4,1-phenylene))bis(methanylylidene))bis(hydrazin-1-yl-2-ylidene))diphthalazine***10b**. Yellow powder in yield 76% (A, reaction time 6 h), 90% (B, 4 min); mp 236–238 °C. IR (KBr): 3291 (NH), 1613, 1528 (C=N), cm^−1^. ^1^H-NMR (DMSO-*d*_6_): δ 1.44 (brs, 4H, 2CH_2_), 1.59 (brs, 2H, CH_2_), 3.57 (brs, 4H, 2N-CH_2_), 7.29 (d, 4H, *J* = 8.8 Hz), 7.65–7.82 (m, 6H), 8.10 (d, 6H, *J* = 9.6 Hz), 8.26 (d, 2H, *J* = 7.2 Hz), 8.45 (s, 2H), 12.15 (s, 2H, NH) ppm; ^13^C-NMR (DMSO-*d*_6_): δ 23.9, 25.1, 44.1, 121.7, 123.6, 126.1, 126.4, 127.0, 129.0, 131.7, 132.3, 132.9, 137.6, 148.5, 152.2, 152.8, 160.1, 165.7, 171.7 ppm. Anal. Calcd for C_38_H_32_N_12_O_2_ (688.28): C, 66.27; H, 4.68; N, 24.40; found: C, 66.41; H, 4.89; N, 24.65. UPLC-MS: calc. m/z: 689.53 (M + 1); found 690.56 (M + 2).

*N,N-Diethyl-4,6-bis(4-((E)-(2-(phthalazin-1-yl)hydrazono)methyl)phenoxy)-1,3,5-triazin-2-amine***10c**. Yellow powder in yield 75% (A, reaction time 5 h), 88% (B, 4 min); mp 217–220 °C; IR (KBr): 3404 (NH), 1615, 1526 (C=N) cm^−1^; ^1^H-NMR (DMSO-*d*_6_): δ 0.97 (t, 6H, *J* = 6.8 Hz, 2CH_3_), 3.32 (m, 4H, 2CH_2_), 7.28 (d, 4H, *J* = 8.8 Hz), 7.72–7.77 (m, 6H), 8.08 (d, 6H, *J* = 8.8 Hz), 8.27 (d, 2H, *J* = 7.6 Hz), 8.46 (s, 2H), 12.15 (s, 2H, NH) ppm; ^13^C-NMR (DMSO-*d*_6_): δ 12.6, 41.5, 121.8, 123.6, 126.1, 126.4, 127.0, 128.9, 131.7, 132.9, 137.6, 148.5, 152.3, 152.9, 165.8, 171.5 ppm; Anal. Calcd for C_37_H_32_N_12_O_2_ (676.75): C, 65.67; H, 4.77; N, 24.84; found: C, 65.91; H, 4.89; N, 25.05. UPLC-MS: calc. m/z: 677.75; found: 679.76 (M + 2).

*4-(4,6-bis(2-Methoxy-4-((E)-(2-(phthalazin-1-yl)hydrazono)methyl)phenoxy)-1,3,5-triazin-2-yl)morpholine***11a**. Yellow powder in yield 77% (A, reaction time 5 h), 92% (B, 4 min); mp 245–247 °C. IR (KBr): 3309 (NH), 1603 (C=O), 1589, 1532 (C=N) cm^−1^; ^1^H-NMR (DMSO-*d*_6_): δ 3.34–3.54 (m, 8H, 4CH_2_), 3.88 (s, 6H, 2O-CH_3_), 7.19 (d, 2H, *J* = 8.4 Hz), 7.41 (d, 2H, *J* = 6.4 Hz, Ar-H), 7.72–7.77 (m, 6H), 7.97 (s, 2H, Ar-H), 8.11 (s, 2H), 8.28 (d, 2H, *J* = 7.6 Hz), 8.43 (s, 2H), 12.24 (s, 2H, NH) ppm; ^13^C-NMR (DMSO-*d*_6_): δ 43.5, 56.1, 65.5, 110.7, 122.5, 123.7, 126.1, 126.5, 127.0, 131.8, 132.3, 134.2, 137.7, 141.7, 148.5, 151.6, 152.5, 165.8, 171.6 ppm. Anal. Calcd for C_39_H_34_N_12_O_5_ (750.28): C, 62.39; H, 4.56; N, 22.39; found: C, 62.64; H, 4.76; N, 22.68.

*1,1′-(((1E,1′E)-(((6-(Piperidin-1-yl)-1,3,5-triazine-2,4-diyl)bis(oxy))bis(3-methoxy-4,1-phenylene))bis(methanylylidene))bis(hydrazin-1-yl-2-ylidene))diphthalazine***11b**. Yellow powder in yield 79% (A, reaction time 6 h), 89% (B, 4 min); mp 215–216 °C. IR (KBr): 3202 (NH), 1654 (C=O), 1584, 1528 (C=N) cm^−1^. ^1^H-NMR (DMSO-*d*_6_): δ 1.41 (brs, 4H, 2CH_2_), 1.53 (brs, 2H, CH_2_), 3.57 (brs, 4H, 2N-CH_2_), 3.88 (s, 6H, 2O-CH_3_), 7.19 (d, 2H, *J* = 8.0 Hz), 7.39 (d, 2H, *J* = 6.4Hz), 7.69–7.77 (m, 6H), 7.91 (s, 2H), 8.11 (s, 2H), 8.27 (d, 2H, *J* = 8.0 Hz,), 8.43 (s, 2H), 12.25 (s, 2H, NH) ppm; ^13^C-NMR (DMSO-*d*_6_): δ 24.1, 25.1, 40.1, 56.2, 108.4, 110.6, 115.6, 121.9, 123.7, 126.1, 126.4, 127.0, 131.8, 134.2, 137.7, 141.8, 148.5, 151.3, 152.6, 165.7, 171.6 ppm. Anal. Calcd for C_40_H_36_N_12_O_4_ (748.30): C, 64.16; H, 4.85; N, 22.45; found: C, 64.35; H, 5.02; N, 22.21. UPLC-MS: calcd. m/z: 748.30; found: 749.91 (M + 1).

*N,N-Diethyl-4,6-bis(2-methoxy-4-((E)-(2-(phthalazin-1-yl)hydrazono)methyl)phenoxy)-1,3,5-triazin-2-amine***11c**. Yellow powder in yield 74% (A, reaction time 4 h), 89% (B, 4 min); mp 228–230 °C. IR (KBr): 3301(NH), 1644 (C=O), 1588 (C=N) cm^−1^. ^1^H-NMR (DMSO-*d*_6_): δ 0.93(t, 6H, *J* = 6.4Hz, 2CH_3_), 3.28–3.50 (m, 4H, 2CH_2_), 3.88 (s, 6H, 2O-CH_3_), 7.21 (d, 2H, *J* = 8.0 Hz), 7.41 (d, 2H, *J* = 6.8 Hz), 7.72–7.77 (m, 6H), 7.97 (s, 2H), 8.12 (s, 2H), 8.28 (d, 2H, *J* = 8.0 Hz), 8.44 (s, 2H), 12.25 (s, 2H, NH) ppm; ^13^C-NMR (DMSO-*d*_6_): δ 12.5, 40.1, 56.1, 110.5, 121.8, 122.6, 123.7, 126.1, 126.5, 127.1, 131.8, 132.3, 134.2, 137.7, 141.8, 148.5, 151.3, 152.6, 165.7, 171.5 ppm. Anal. Calcd for C_39_H_36_N_12_O_4_ (736.30): C, 63.58; H, 4.93; N, 22.81; found: C, 63.77; H, 5.16; N, 23.06. UPLC-MS: calcd. m/z: 736.30; found: 737.91 (M + 1), 738.85 (M + 2).

### 3.4. General Method for the Reaction of Dipodal ***3***–***8*** with Barbiturate Derivatives

**Method A:** Conventional heating

The barbituric acid derivative **12** or **13** (4 mmol) was added to a solution of dipodal derivatives **3**–**8** (2 mmol) in 10 mL of absolute ethanol, mixed well at room temperature for 5 min, and then refluxed for 4–6 h until the reaction was completed (TLC:ethyl acetate hexane, 6:4; CHCl_3_:MeOH, 9:1). The solvent was evaporated under reduced pressure and the crude solid crystallized from DCM–hexane to afford the pure product.

**Method B:** Microwave irradiation

The dipodal derivatives **3**–**8** (2 mmol) and barbituric acid derivative **12** or **13** (4 mmol) in 5 mL of EtOH were mixed at room temperature and then irradiated in a microwave oven for 4 min. After cooling, the resulting solid was recrystallized from DCM–hexane to afford the pure product.

*5,5′-((((6-Morpholino-1,3,5-triazine-2,4-diyl)bis(oxy))bis(4,1-phenylene))bis(methanylylidene))bis(1,3-dimethylpyrimidine-2,4,6(1H,3H,5H)-trione)***14**. Yellow powder in yield 81% (A, 5 h). 90% (B, 4 min), mp 204–206 °C. ^1^H-NMR (CDCl_3_): δ 3.35 (brs, 4H, N-CH_2_), 3.45 (brs, 4H, O-CH_2_), 3.67 (s, 12H, 4CH_3_), 7.21 (d, 4H, *J* = 7.6 Hz); 8.17 (d, 4H), 8.47 (s, 2H, CH=C) ppm; ^13^C-NMR (CDCl_3_): δ 29.1, 44.1, 66.4, 116.9, 121.6, 129.8, 135.7, 151.1, 155.4, 157.8, 160.0, 166.7, 171.2 ppm. Anal. Calcd for C_33_H_30_N_8_O_9_ (682.21): C, 58.06; H, 4.43; N, 16.4; found: C, 58.29; H, 4.63; N, 16.69.

*5,5′-((((6-(Diethylamino)-1,3,5-triazine-2,4-diyl)bis(oxy))bis(4,1-phenylene))bis(methanylylidene))bis(1,3-dimethylpyrimidine-2,4,6(1H,3H,5H)-trione)***15**. Yellow powder in yield 82% (A, 4 h), 92% (B, 4 min); mp 224–245 °C. ^1^H-NMR (CDCl_3_): δ 1.10–1.13 (m, 6H, 2CH_3_, ethyl residue), 3.38–3.41 (m, 12H, 4N-CH_3_), 4.49 (s, 4H, 2N-CH_2_, ethyl residue), 7.26 (d, 4H, *J* = 8 Hz); 8.2 (d, 4H, *J* = 8.8 Hz), 8.49 (s, 2H, CH=C) ppm; ^13^C-NMR (CDCl_3_): δ 12.8, 29.1, 42.2, 116.8, 121.8, 129.7, 135.7, 155.7, 158.0, 160.5, 162.6, 166.2, 171.4 ppm. Anal. Calcd for C_33_H_32_N_8_O_8_ (668.23): C, 59.28; H, 4.82; N, 16.76, found: C, 59.55; H, 4.96; N, 16.98.

*5,5′-((((6-Morpholino-1,3,5-triazine-2,4-diyl)bis(oxy))bis(3-methoxy-4,1-phenylene))bis(methanylylidene))bis(1,3-dimethylpyrimidine-2,4,6(1H,3H,5H)-trione)***16**. Yellow powder in yield 79% (A, 6 h), 91% (B, 4 min), mp 252–253 °C. ^1^H-NMR (CDCl_3_): δ 3.36 (s, 12H, 4N-CH_3_), 3.69 (brs, 4H, 2N-CH_2_), 3.77 (brs, 10H, 2O-CH_2_, 2O-CH_3_), 7.05 (d, 2H, *J* = 8.0 Hz); 7.54 (s, 2H), 7.97 (d, 2H, *J* = 1.6 Hz), 8.30 (s, 2H, CH=C) ppm; ^13^C-NMR (CDCl_3_): δ 29.0, 44.1, 55.9, 66.5, 116.6, 117.5, 122.2, 128.7, 130.8, 145.0, 150.9, 151.1, 158.0, 160.0, 162.6, 167.7, 171.2 ppm. Anal. Calcd for C_35_H_34_N_8_O_11_ (742.23): C, 58.66; H, 4.61; N, 15.09; found: C, 58.49; H, 4.86; N, 15.28.

*5,5′-((((6-(Diethylamino)-1,3,5-triazine-2,4-diyl)bis(oxy))bis(3-methoxy-4,1-phenylene))bis(methanylylidene))bis(1,3-dimethylpyrimidine-2,4,6(1H,3H,5H)-trione)***17**. The product obtained as a yellow powder in yield 83% (A, 5 h), 92% (B, 4 min), mp 249–251 °C. ^1^H-NMR (CDCl_3_): δ 1.07–1.10 (m, 6H, 2CH_3_), 3.37 (m, 12H, 4N-CH_3_), 3.45–3.48 (m, 4H, 2N-CH_2_), 3.79–3.82 (m, 6H, 2O-CH_3_), 7.13 (d, 2H, *J* = 8.4 Hz); 7.60 (dd, 2H, *J* = 2 Hz, *J* = 8.8 Hz), 8.04 (d, 2H, *J* = 1.6 Hz), 8.36(s, 2H, CH=C) ppm; ^13^C-NMR (CDCl_3_): δ 12.8, 29.0, 42.0, 55.9, 116.5, 117.5, 122.5, 128.9, 130.7, 145.4, 151.2, 158.2, 160.5, 162.6, 166.5, 171.2 ppm. Anal. Calcd for C_35_H_36_N_8_O_10_ (728.26): C, 57.69; H, 4.98; N, 15.38; found: C, 57.90; H, 5.16; N, 15.56.

*5,5′-((((6-Morpholino-1,3,5-triazine-2,4-diyl)bis(oxy))bis(4,1-phenylene))bis(methanylylidene))bis(1,3-diethyl-2-thioxodihydropyrimidine-4,6(1H,5H)-dione)***18**. Yellow powder in yield 86% (A, 5 h), 92% (B, 4 min); mp 221–223 °C. ^1^H-NMR (CDCl_3_): δ 1.28–1.32 (m, 12H, 4CH_3_), 3.68–3.73 (m, 8H, 2N-CH_2_CH_2_-O morpholine residue), 4.57 (m, 8H, 4N-CH_2_), 7.26 (d, 4H, *J* = 8.8 Hz); 8.23 (d, 4H, *J* = 8.8 Hz), 8.47 (s, 2H, CH=C) ppm; ^13^C-NMR (CDCl_3_): δ 12.3, 43.6, 44.1, 66.4, 117.6, 121.7, 130.1, 135.98, 155.6, 158.8, 160.6, 166.6, 171.6, 178.7 ppm. Anal. Calcd for C_37_H_38_N_8_O_7_S_2_ (770.23): C, 57.65; H, 4.97; N, 14.54; found: C, 57.93; H, 5.12; N, 14.77.

*5,5′-((((6-Morpholino-1,3,5-triazine-2,4-diyl)bis(oxy))bis(3-methoxy-4,1-phenylene))bis(methanylylidene))bis(1,3-diethyl-2-thioxodihydropyrimidine-4,6(1H,5H)-dione)***19**. Yellow powder in yield 73% (A, 5 h), 90% (B 4 min); mp 195–196 °C. ^1^H-NMR (CDCl_3_): δ 1.22–1.29 (m, 12H, 4CH_3_), 3.67 (m, 6H, 2O-CH_3_), 3.77 (m, 8H, 4N-CH_2_), 4.55 (m, 8H, 2N-CH_2_CH_2_-O), 7.1 (d, 2H, *J* = 8.8 Hz); 7.62 (d, 2H, *J* = 8.4 Hz), 7.99 (s, 2H), 8.34 (s, 2H, CH=C) ppm; ^13^C-NMR (CDCl_3_): δ 12.3, 43.6, 44.2, 55.9, 66.5, 117.5, 122.4, 128.7, 131.1, 145.1, 150.9, 151.1, 158.5, 160.0, 166.9, 171.5, 178.8 ppm. Anal. Calcd for C_39_H_42_N_8_O_9_S_2_ (830.23): C, 56.37; H, 5.09; N, 13.49; found: C, 56.61; H, 5.28; N, 13.16.

*5,5′-((((6-(Piperidin-1-yl)-1,3,5-triazine-2,4-diyl)bis(oxy))bis(3-methoxy-4,1-phenylene))bis(methanylylidene))bis(1,3-diethyl-2-thioxodihydropyrimidine-4,6(1H,5H)-dione)***20**. Yellow powder in yield 80% (A, 4 h), 89% (B, 4 min); mp 197–198 °C. ^1^H-NMR (CDCl_3_): δ 1.28 (t, 12H, *J* = 6.4 Hz, 4CH_3_), 1.48 (brs, 4H, 2CH_2_), 1.62 (brs, 2H, CH_2_), 3.69 (brs, 4H, 2N-CH_2_), 1.82 (s, 6H, 2O-CH_3_), 4.55 (m, 8H, 4CH_2_), 7.1 (d, 2H, *J* = 8.8 Hz); 7.62 (d, 2H, *J* = 8.4 Hz), 8.01 (s, 2H), 8.35 (s, 2H, CH=C) ppm; ^13^C-NMR (CDCl_3_): δ 12.4, 24.5, 25.7, 43.6, 44.2, 45.0, 55.9, 117.6, 122.5, 128.9, 131.1, 145.4, 151.0, 158.6, 159.1, 160.9, 171.2, 178.8 ppm. Anal. Calcd for C_40_H_44_N_8_O_8_S_2_ (828.96): C, 57.96; H, 5.35; N, 13.52; found: C, 57.74; H, 5.21; N, 13.78.

*5,5′-((((6-(Diethylamino)-1,3,5-triazine-2,4-diyl)bis(oxy))bis(4,1-phenylene))bis(methanylylidene))bis(1,3-diethyl-2-thioxodihydropyrimidine-4,6(1H,5H)-dione)***21**. Yellow powder in yield 82% (A, 6 h), 89% (B, 4 min); mp 215–217 °C. ^1^H-NMR (CDCl_3_): δ 1.04–1.08 (m, 6H, 2CH_3_), 3.41–3.45 (m, 12H, 4N-CH_3_), 3.45–3.48 (m, 4H, 2N-CH_2_), 3.81 (s, 6H, 2O-CH_3_), 4.49–4.54 (s, 8H, 4N-CH_2_), 7.13 (d, 2H, *J* = 8.8 Hz); 7.60 (d, 2H, *J* = 10 Hz), 8.06 (d, 2H, *J* = 2.0 Hz), 8.38 (s, 2H) ppm; ^13^C-NMR (CDCl_3_): δ 12.7, 42.1, 44.2, 56.0, 116.5, 117.5, 122.6, 129.0, 131.0, 145.5, 151.1, 158.5, 161.0, 162.6, 171.2, 179.8 ppm. Anal. Calcd for C_39_H_44_N_8_O_8_S_2_ (816.27): C, 57.34; H, 5.43; N, 13.72; found: C, 58.57; H, 5.56; N, 14.00.

## 4. Conclusions

Due to the different reactivities of the three chlorine atoms in cyanuric chloride, these chlorine atoms could be replaced using different nucleophiles by controlling the temperature. Moreover, cyanuric chloride also showed good selectivity toward substitution by the amino-to-hydroxyl group. We used this advantage for the preparation of a novel series of di-arm *s*-triazine derivatives (dipodal). These di-arm aldehyde derivatives were used for the preparation of a new series of oxy-Schiff bases, oxybenzylidene barbituric and thiobarbituric acid derivatives, using conventional heating or microwave irradiation. Microwave irradiation was usually done in less solvent and afforded the target products in shorter reaction time, with higher yields and purities. NMR (^1^H and ^13^C), elemental analyses and UPLC-MS confirmed the structures of the prepared compounds. 

Efforts made on the synthesis and characterization of higher generation of *s*-triazine derivatives are in progress in our lab, which might be of special interest in medicinal chemistry and coordination chemistry for other researchers.

## Supplementary Materials

Spectral data are available online: ^1^H-NMR, ^13^C-NMR, and UPLC-MS.

## References

[1] Giguere R.J., Bray T.L., Duncan S.M., Majetich G. (1986). Application of commercial microwave ovens to organic synthesis. Tetrahedron Lett..

[2] Stadler A., Kappe C.O., Lidström P., Tierny J.P. (2005). Microwave- Assisted Organic Synthesis.

[3] Caddick S. (1995). Microwave assisted organic reactions. Tetrahedron.

[4] Hayes B.L. (2004). Recent advances in microwave assisted synthesis. Aldrichim. Acta.

[5] Moseley J.D., Kappe C.O. (2011). A critical assessment of the greenness and energy efficiency of microwave-assisted organic synthesis. Green Chem..

[6] Kappe C.O., Dallinge D. (2009). Controlled microwave heating in modern organic synthesis: Highlights from the 2004–2008. Mol. Divers..

[7] Kappe C.O., Damm M. (2012). Parallel microwave chemistry in silicon carbide microtiter platforms: A review. Mol. Divers..

[8] Zhu W., Liu Y., Zhao Y., Wang H., Tan L., Fan W., Gong P. (2012). Synthesis and biological evaluation of novel 6-hydrazinyl-2,4-bismorpholino pyrimidine and 1,3,5-triazine derivatives as potential antitumor agents. Arch. Pharm..

[9] Iino Y., Karakida T., Sugamata N., Andoh T., Takei H., Takahashi M., Yaguchi S., Matsuno T., Takehara M., Sakato M. (1998). Antitumor effects of SEF19, a new nonsteroidal aromatase inhibitor, on 7,12-dimethyl benz[a]anthracene-induced mammary tumors in rats. Anticancer Res..

[10] Matsuno T., Kato M., Sasahara H., Watanbe T., Inaba M., Takahashi M., Yaguchi S.-I., Yoshioka K., Sakato M., Kawashima S. (2000). Synthesis and antitumor activity of benzimidazolyl-1, 3, 5-triazine and benzimidazolyl pyrimidine derivatives. Chem. Pharm. Bull..

[11] Smolin E.M., Rapoport L. (2008). Chemistry of Heterocyclic Compounds: s-Triazines and Derivatives.

[12] Giacomelli G., Porcheddu A., Turnbull K. (2008). Comprehensive Heterocyclic Chemistry III.

[13] Blotny G. (2006). Recent applications of 2,4,6-trichloro-1,3,5-triazine and its derivatives in organic synthesis. Tetrahedron.

[14] Brzozowski Z., Sączewski F. (2002). Synthesis and antitumor activity of novel 2-amino-4-(3,5,5-trimethyl-2-pyrazolino)-1,3,5-triazine derivatives. Eur. J. Med. Chem..

[15] Desai N.C., Makwana A.H., Senta R.D. (2016). Synthesis, characterization and antimicrobial activity of some novel 4-(4-(arylamino)-6-(piperidin-1-yl)-1,3,5-triazine-2-ylamino)-*N*-(pyrimidin-2-yl)benzene sulfonamides. J. Saudi Chem. Soc..

[16] Khan F.G., Yadav M.V., Sagar A.D. (2014). Synthesis, characterization, and antimicrobial evaluation of novel trichalcones containing core s-triazine moiety. Med. Chem. Res..

[17] Freeman A.W., Vreekamp R., Fréchet J.M.J. (1997). Book of Abstracts. Proceedings of the 214th ACS National Meeting, Las Vegas, NV, USA, 7–11 September 1997.

[18] Afonso C.A.M., Lourenço N.M.T., Rosatella A.A. (2002). Synthesis of 2,4,6-tri-substituted-1,3,5-triazines. Molecules.

[19] Cho S.Y., Chang Y., Kim J.S., Lee S.C., Kim C. (2001). Dendrimers based on [1,3,5]-triazines. Macromol. Chem. Phys..

[20] Kataoka Y., Kondo T. (1996). Changing cellulose crystalline structure in forming wood cell walls. Macromolecules.

[21] Steffensen M.B., Hollink E., Kuschel F., Bauer M., Simanek E.E. (2006). Dendrimers based on [1,3,5]-triazines. J. Polym. Sci. A Polym. Chem..

[22] White W., Hudson Z.M., Feng X.-D., Han S., Lu Z.-H., Wang S. (2010). Linear and star-shaped benzimidazolyl derivatives: Syntheses, photophysical properties and use as highly efficient electron transport materials in OLEDs. Dalton Trans..

[23] Machakanur S.S., Patil B.R., Badiger D.S., Bakale R.P., Gudasi K.B., Bligh S.W.A. (2012). Synthesis, characterization and anticancer evaluation of novel tri-arm star shaped 1,3,5-triazine hydrazones. J. Mol. Str..

[24] Tahmassebi D.C., Sasaki T.J. (1994). Synthesis of a new trialdehyde template for molecular imprinting. J. Org. Chem..

[25] Koc Z.E., Ucan H.I. (2007). Complexes of iron(III) salen and saloph Schiff bases with bridging 2,4,6-*tris*(2,5-dicarboxyphenylimino-4-formylphenoxy)-1,3,5-triazine and 2,4,6-*tris*(4-carboxyphenylimino-4′-formylphenoxy)-1,3,5-triazine. Trans. Metal. Chem..

[26] Koc Z.E., Bingol H., Saf A.O., Torlak E., Coskun A. (2010). Synthesis of novel tripodal-benzimidazole from 2, 4, 6-tris(p-formylphenoxy)-1,3,5-triazine: Structural, electrochemical and antimicrobial studies. J. Hazard. Mater..

[27] Celikbilek S., Koc Z.E. (2014). Investigation of Dipodal oxy-Schiff base and its salen and salophen Fe(III)/Cr(III)/Mn(III) Schiff bases (N_2_O_2_) caped complexes and their magnetic and thermal behaviors. J. Mol. Struct..

[28] Jarrahpour A., Khalili D., De Clercq E., Salmi C., Brunel J.M. (2007). Synthesis, antibacterial, antifungal and antiviral activity evaluation of some new bis-Schiff bases of isatin and their derivatives. Molecules.

[29] Hameed A., Al-Rashida M., Uroos M., Ali A.S., Khan K.M. (2017). Schiff bases in medicinal chemistry: A patent review (2010-2015). Expert Opin. Ther. Patents.

[30] Sinha D., Tiwari A.K., Singh S., Shukla G., Mishra P., Chandra H., Mishra A.K. (2008). Synthesis, characterization and biological activity of Schiff base analogues of indole-3-carboxaldehyde. Eur. J. Med. Chem..

[31] Singh M., Raghav N. (2011). Biological activities of hydrazones: A review. Int. J. Pharm. Pharmaceut. Sci..

[32] Rollas S., Küçükgüzel S.G. (2007). Biological activities of hydrazone derivatives. Molecules.

[33] Soliman S.M., El-Faham A. (2018). Low temperature X-ray structure analyses combined with NBO studies of a new heteroleptic octa-coordinated Holmium(III) complex with *N*,*N*,*N*-tridentate hydrazono-phthalazine-type ligand. J. Mol. Struct..

[34] Soliman S.M., Albering J.H., Farooq M., Wadaan M.A.M., El-Faham A. (2017). Synthesis, structural and biological studies of two new Co(III) complexes with tridentate hydrazone ligand derived from the antihypertensive drug hydralazine. Inorg. Chim. Acta.

[35] Uhlmann C., Fröscher W. (2009). Low risk of development of substance dependence for barbiturates and clobazam prescribed as antiepileptic drugs: Results from a questionnaire study. CNS Neurosci. Ther..

[36] Breyholz H.J., Wagner S., Faust A., Riemann B., Höltke C., Hermann S., Schober O., Schäfers M., Kopka K. (2010). Radiofluorinated pyrimidine-2,4,6-triones as molecular probes for noninvasive MMP-targeted imaging. Chem. Med. Chem..

[37] Seeliger F., Berger S.T.A., Remennikov G.Y., Polborn K., Mayr H. (2007). Electrophilicity of 5-benzylidene-1,3-dimethylbarbituric and -thiobarbituric acids. J. Org. Chem..

[38] Figueroa-Villar J.D., Oliveira S.C.G. (2011). Synthesis and mechanism of formation of oxadeazaflavines by microwave thermal cyclization of *ortho*-halobenzylidene barbiturates. J. Braz. Chem. Soc..

[39] Zhang W., Jiang J., Qin C., Thomson L.M., Parrish A.R., Safe S.H., Simanek E.E. (2003). Triazine Dendrimers for Drug Delivery: Evaluation of solubilization properties, activity in cell culture, and in vivo toxicity of a candidate vehicle. Supramol. Chem..

[40] Lim J., Simanek E.E. (2012). Triazine dendrimers as drug delivery systems: From synthesis to therapy. Adv. Drug Deliv. Rev..

[41] Sharma A., Ghabbour H., Khan S.T., de la Torre B.G., Albericio F., El-Faham A. (2017). Novel pyrazolyl-s-triazine derivatives, molecular structure and antimicrobial activity. J. Mol. Struct..

[42] Padalkar V.S., Patil V.S., Nagaiyan S. (2011). Synthesis of novel fluorescent 1,3,5-trisubstituted triazine derivatives and Photophysical property evaluation of fluorophores and its BSA. Chem. Cent. J..

[43] Barakat A., Al-Majid A.M., Soliman S.M., Lotfy G., Ghabbour H.A., Fun H.-K., Wadood A., Warad I., Sloop J.C. (2015). New diethyl ammonium salt of thiobarbituric acid derivative: Synthesis, molecular structure investigations and docking studies. Molecules.

[44] Barakat A., Al-Majid A.M., Lotfy G., Arshad F., Yousuf S., Choudhary M.I., Ashraf S., Ul-Haq Z. (2015). Synthesis and dynamics studies of barbituric acid derivatives as urease inhibitors. Chem. Cent. J..

[45] Jursic B.S. (2001). A simple method for knoevenagel condensation of α,β-conjugated and aromatic aldehydes with barbituric acid. J. Heterocycl. Chem..

[46] Kim E.G. (2008). Method for Preparing Fluorescent Materials Containing Triazine Groups. Repub. Korea Patent.

[47] Wang S.-J., Lu Z.-R., Dong X., Chen B., Hua C.-W., Gou X.-F., Zhao J.-L. (2015). Synthesis and recognition of metal ions of Schiff base macrocyclic compounds of 1,3,5-triazine. Chin. J. Org. Chem..

[48] Li X., Hua C., Gou X., Zhao J., Chen B. (2012). Synthesis and characterization of novel Schiff base macrocyclic compounds of 1,3,5-triazine. Chin. J. Org. Chem..

