# Supplementary material for: Microwave Irradiation Assists the Synthesis of a Novel Series of bis-Arm s-Triazine Oxy-Schiff Base and Oxybenzylidene Barbiturate Derivatives
Source: Molecules. 2018 Nov 14;23(11):2976. doi: 10.3390/molecules23112976 (PMC6278277; doi:10.3390/molecules23112976)

Supporting Information

Microwave Irradiation Assists the Synthesis of a Novel Series of *bis*-Arm *s*-Triazine Oxy-Schiff Base and Oxybenzylidene Barbiturate Derivatives

Kholood A. Dahlous, Zainab Almarhoon, Ahmed-Yacine Badjah-Hadj-Ahmed, Zeid A. AL Othman, Ayman El-Faham *

Table of content

^{1}H and ^{13}C NMR for Compound 3 **S1**

^{1}H and ^{13}C NMR for Compound 4 **S2**

^{1}H and ^{13}C NMR for Compound 5 **S3**

^{1}H and ^{13}C NMR for Compound 6 **S4**

^{1}H and ^{13}C NMR for Compound 7 **S5**

^{1}H and ^{13}C NMR for Compound 8 **S6**

^{1}H and ^{13}C NMR for Compound 9a **S7**

UPLC-MC compound 9a **S8**

^{1}H and ^{13}C NMR for Compound 9b **S9**

UPLC-MS for compound 9b **S10**

^{1}H and ^{13}C NMR for Compound 9c **S11**

UPLC-MS for compound 9c **S12**

^{1}H and ^{13}C NMR for Compound 10a **S13**

^{1}H and ^{13}C NMR for Compound 10b **S14**

UPLC-MS for compound 10b **S15**

^{1}H and ^{13}C NMR for Compound 10c **S16**

UPLC-MS for compound 10c **S17**

^{1}H and ^{13}C NMR for Compound 11a **S18**

^{1}H and ^{13}C NMR for Compound 11b **S19**

UPLC-MS for compound 11b **S20**

^{1}H and ^{13}C NMR for Compound 11c **S21**

UPLC-MS for compound 11c **S22**

^{1}H and ^{13}C NMR for Compound 14 **S23**

^{1}H and ^{13}C NMR for Compound 15 **S24**

^{1}H and ^{13}C NMR for Compound 16 **S25**

^{1}H and ^{13}C NMR for Compound 17 **S26**

^{1}H and ^{13}C NMR for Compound 18 **S27**

^{1}H and ^{13}C NMR for Compound 19 **S28**

^{1}H and ^{13}C NMR for Compound 20 **S29**

^{1}H and ^{13}C NMR for Compound 21 **S30**

S1

^{1}H and ^{13}C NMR for Compound 3

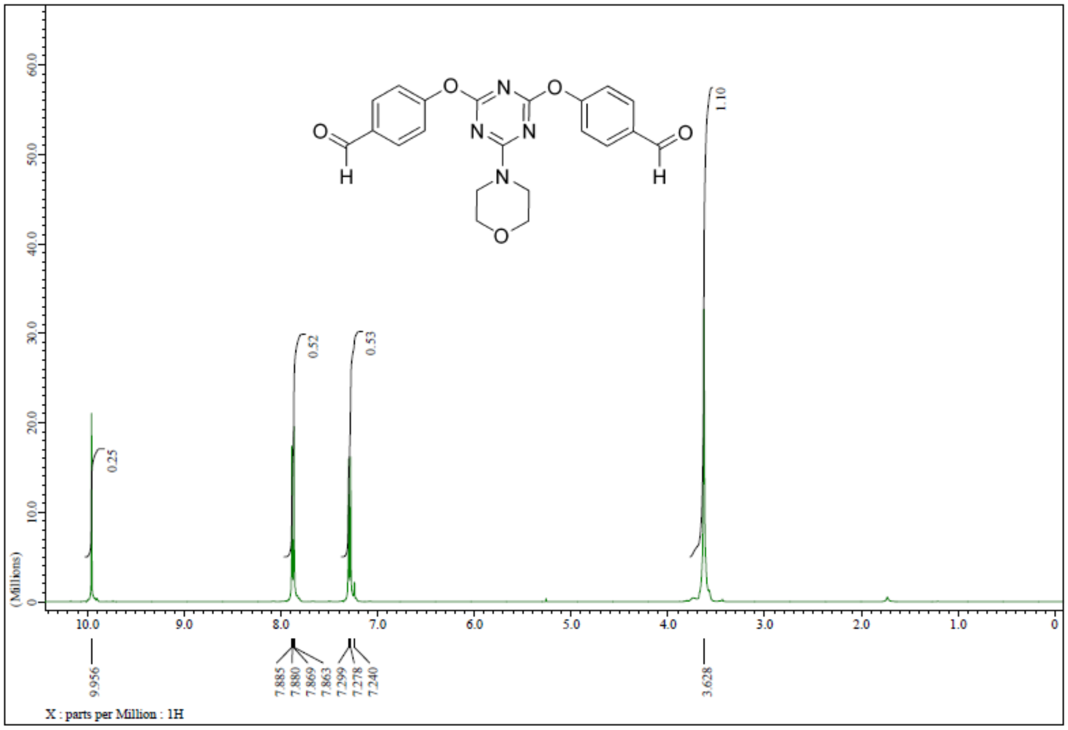

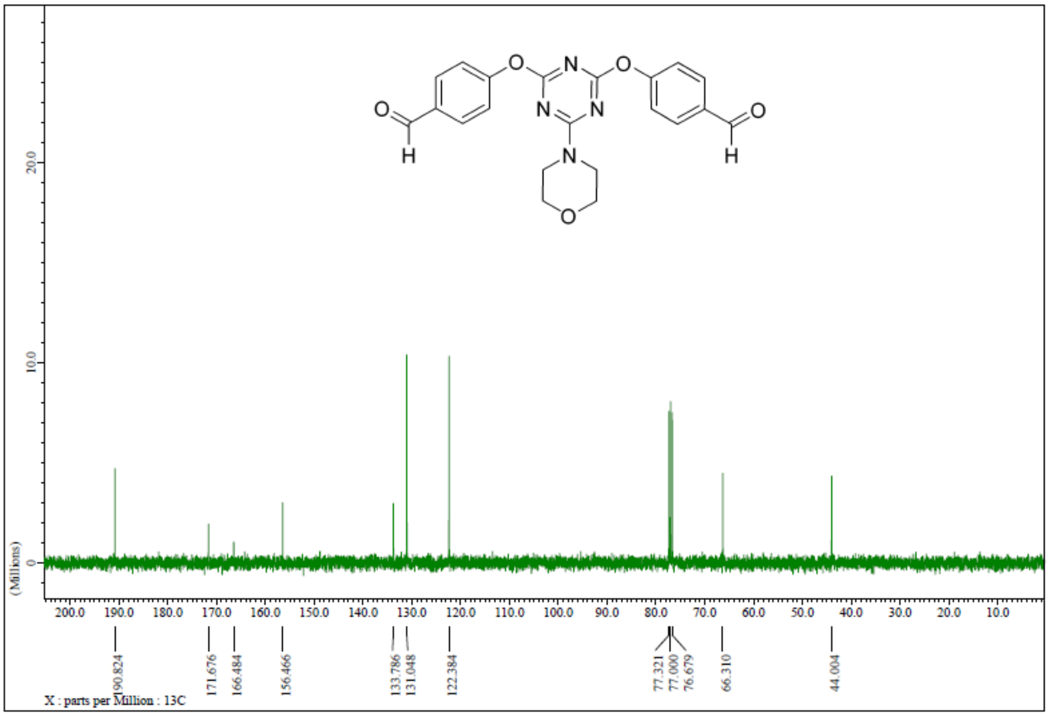

S2

^{1}H and ^{13}C NMR for Compound 4

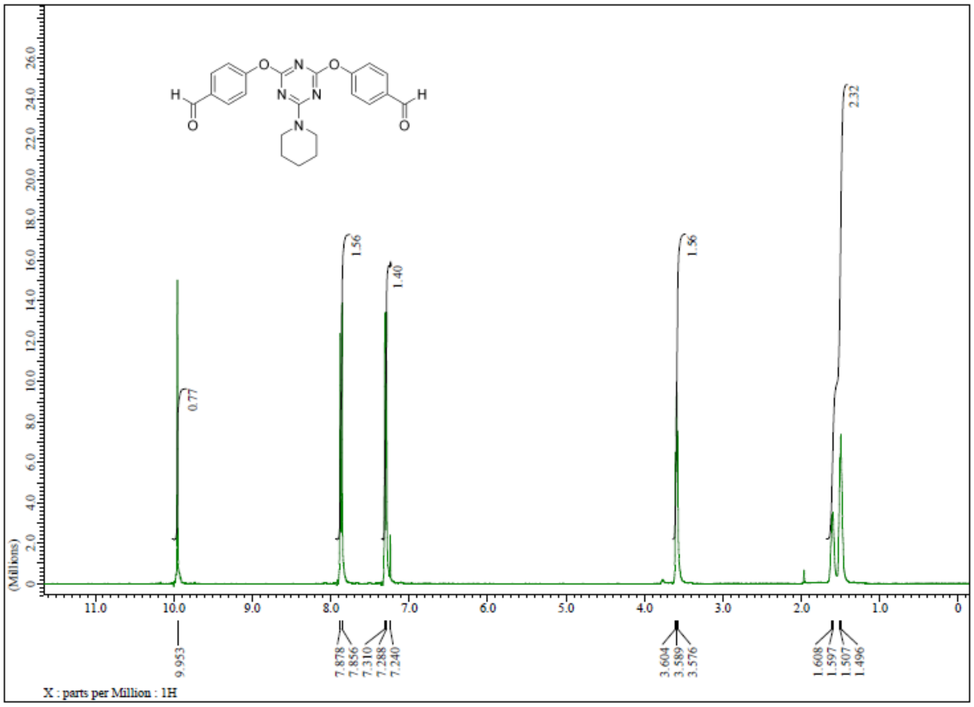

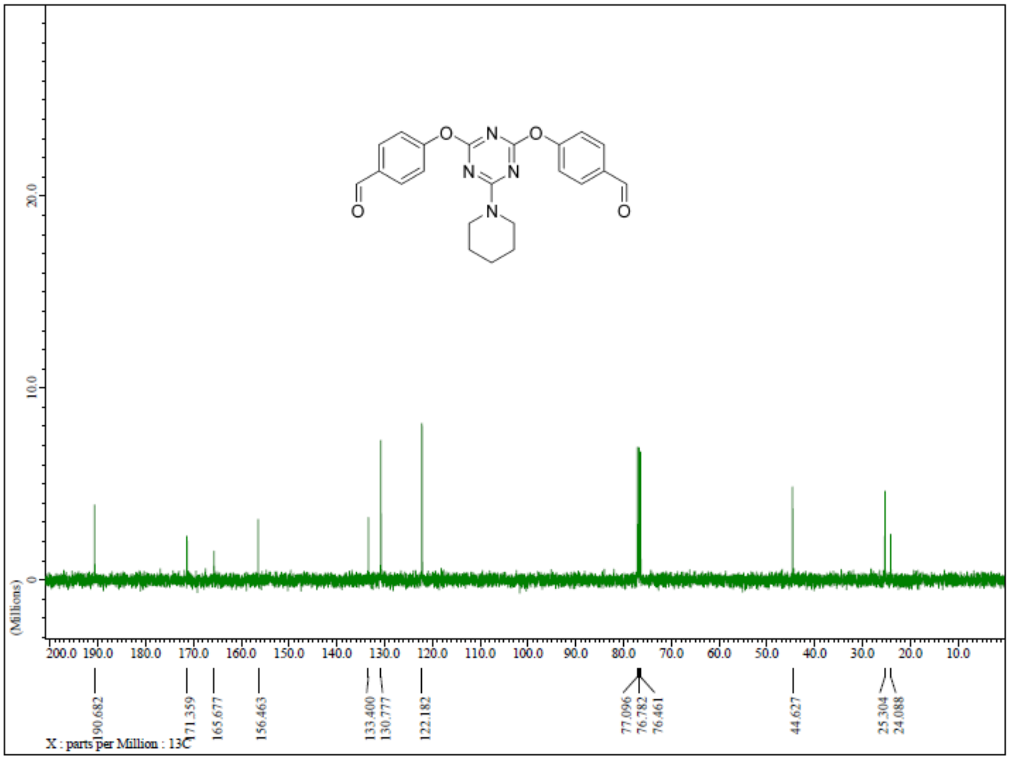

S3

^{1}H and ^{13}C NMR for Compound 5

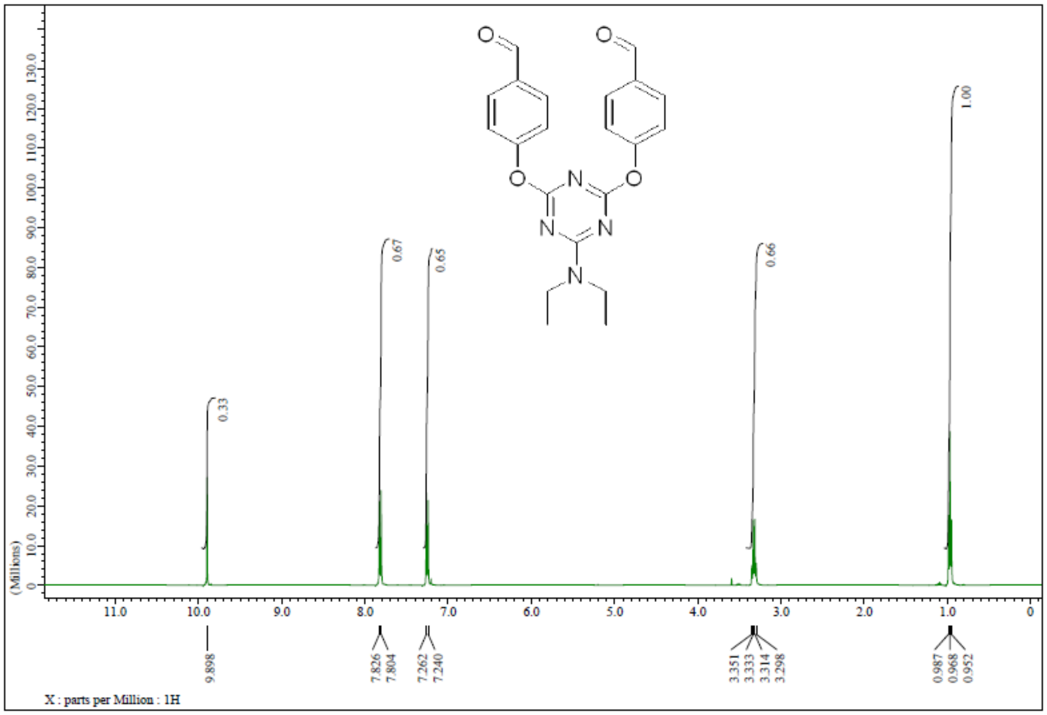

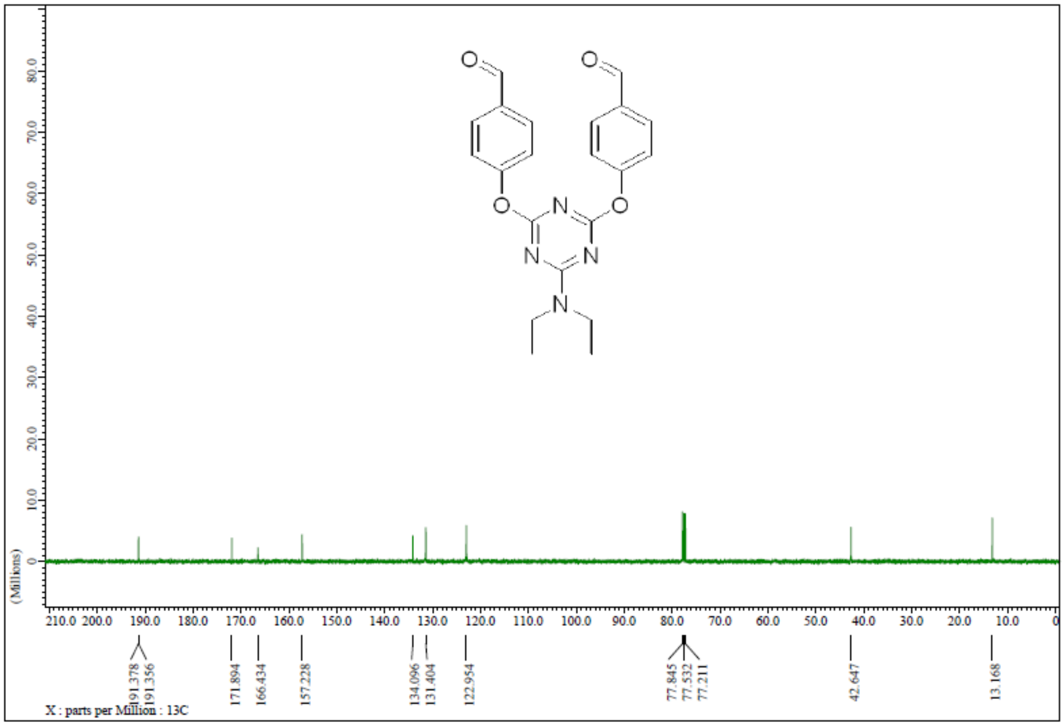

S4

^{1}H and ^{13}C NMR for Compound 6

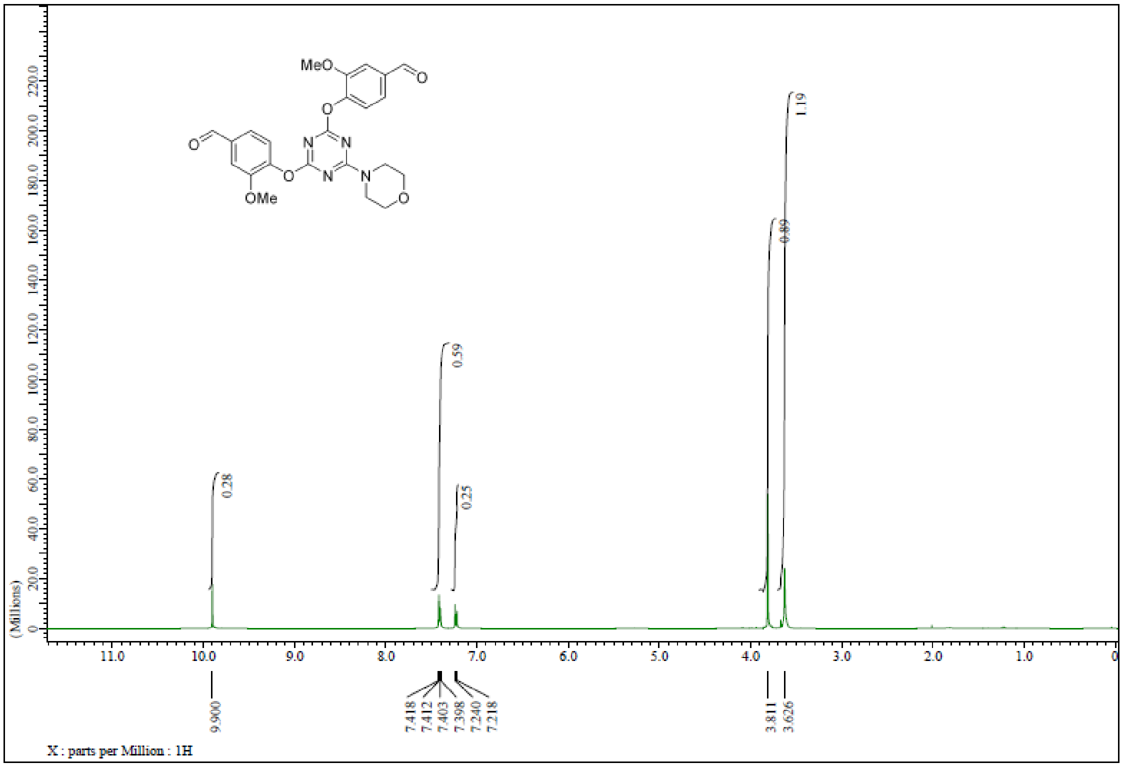

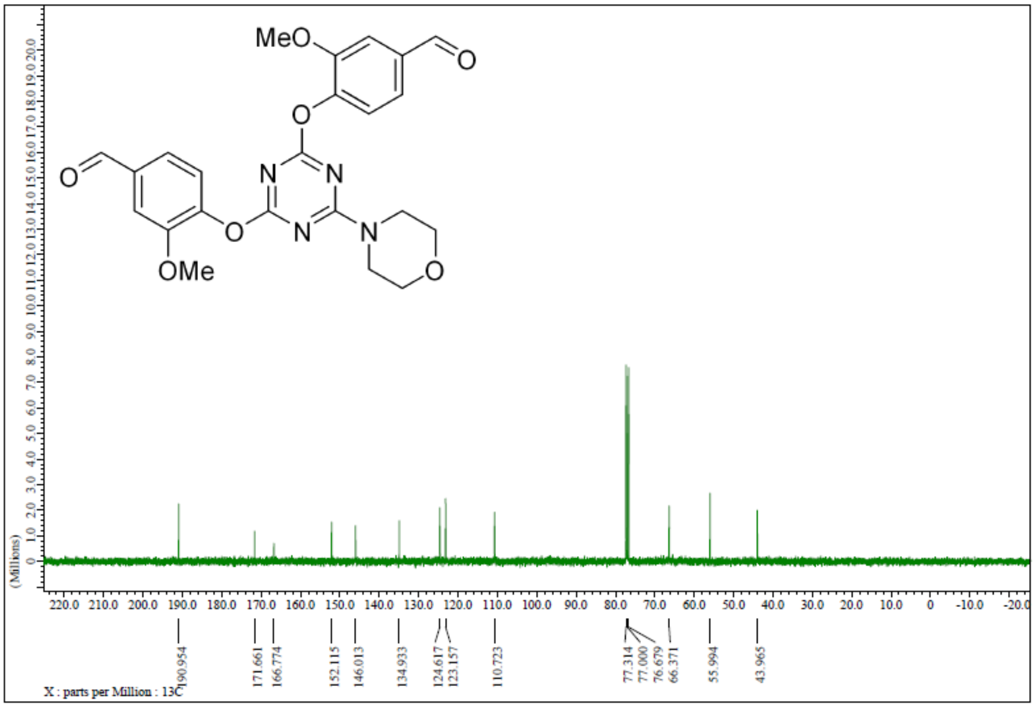

S5

^{1}H and ^{13}C NMR for Compound 7

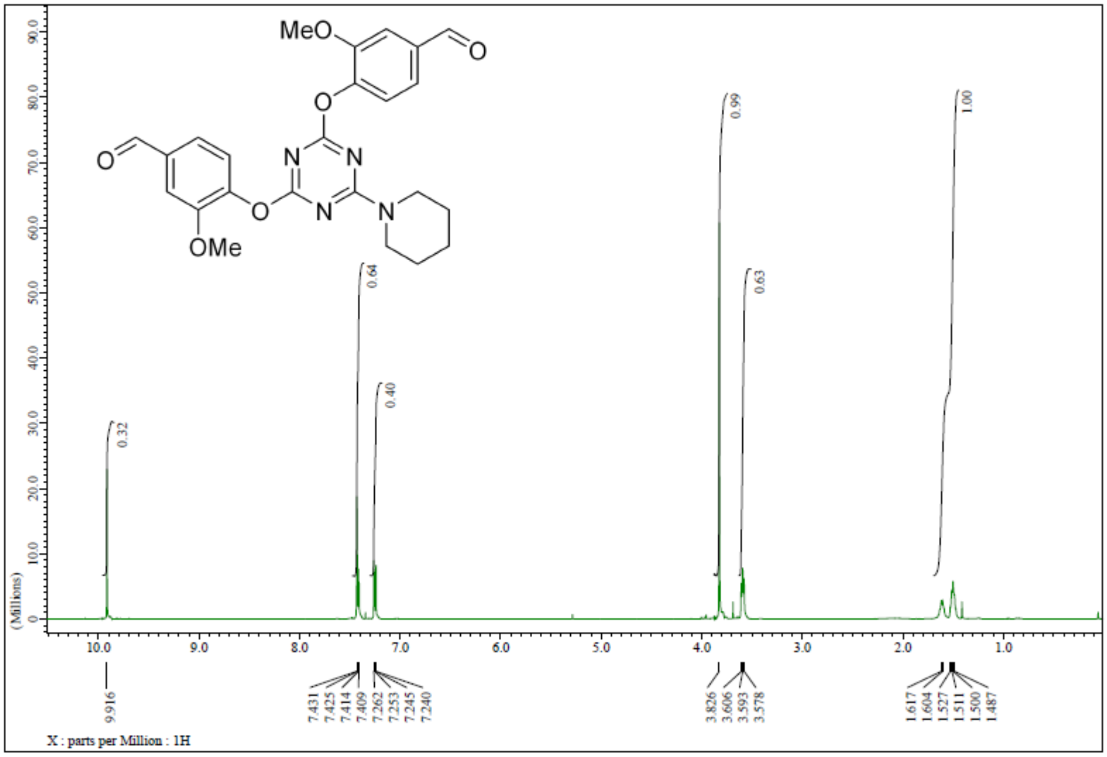

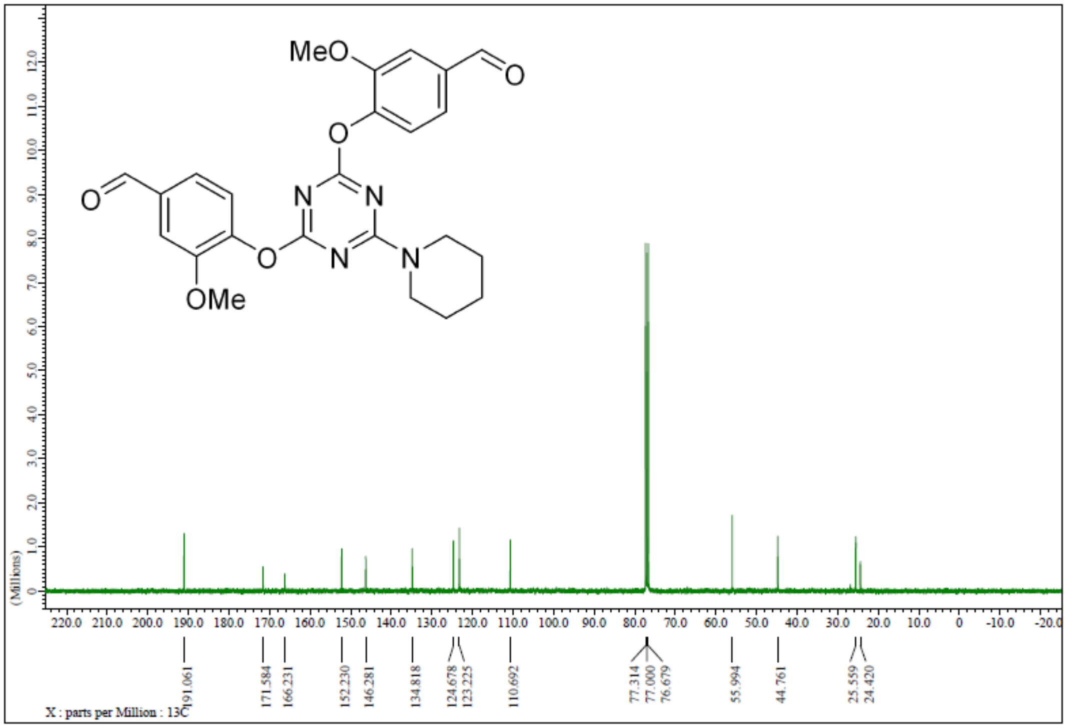

S6

^{1}H and ^{13}C NMR for Compound 8

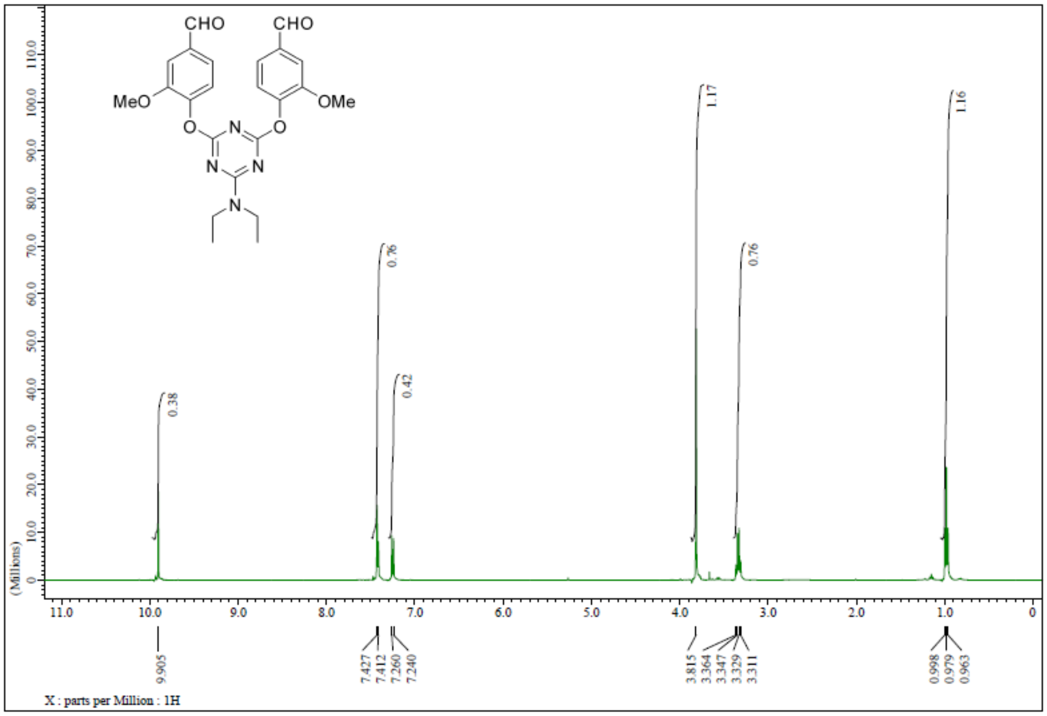

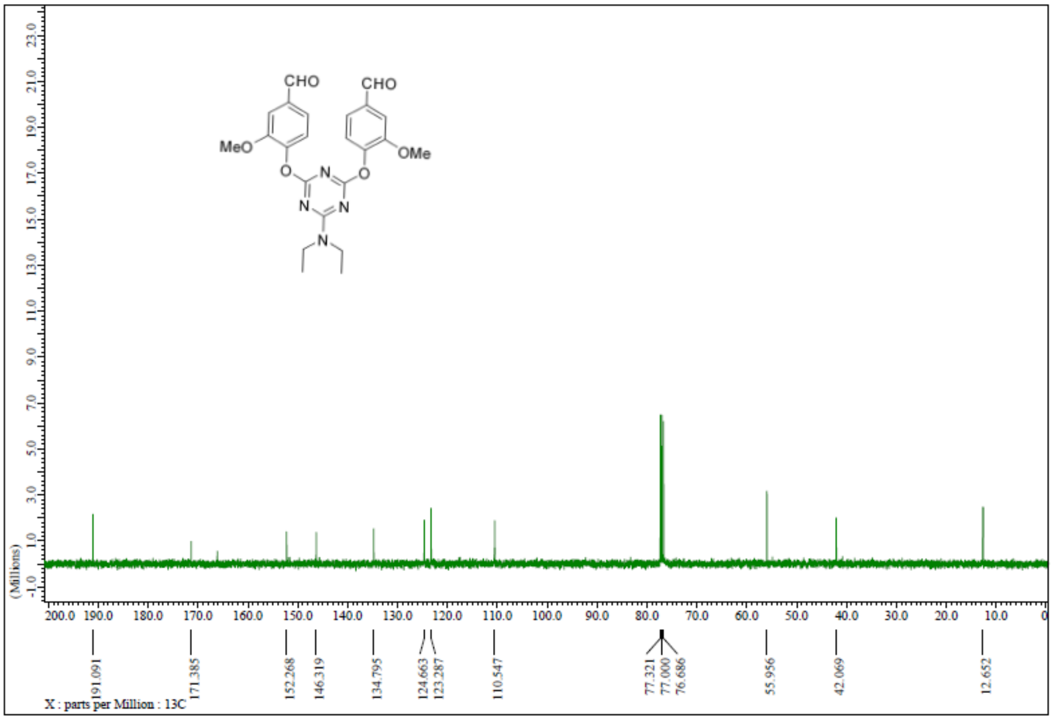

S7

^{1}H and ^{13}C NMR for Compound 9a

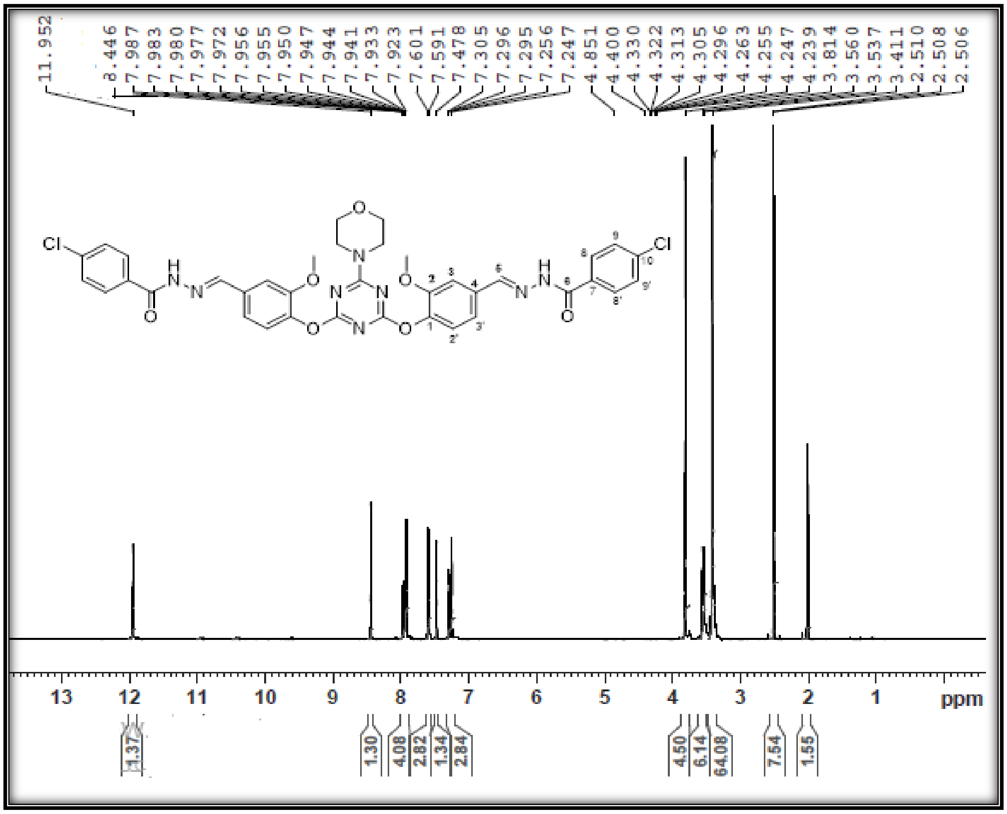

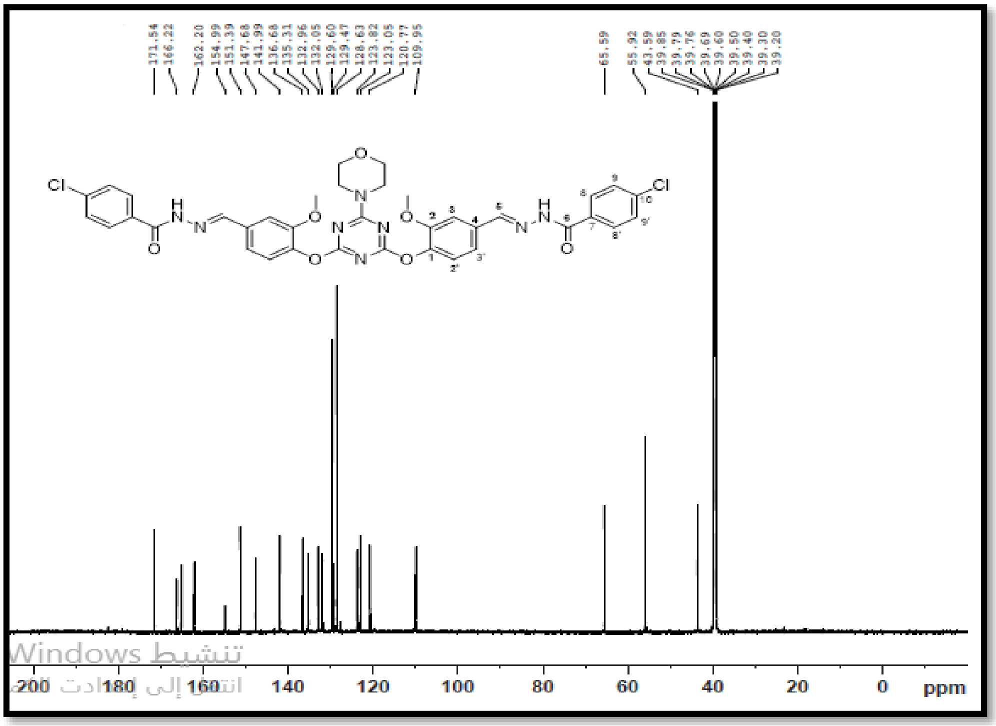

S8

UPLC-MC compound 9a

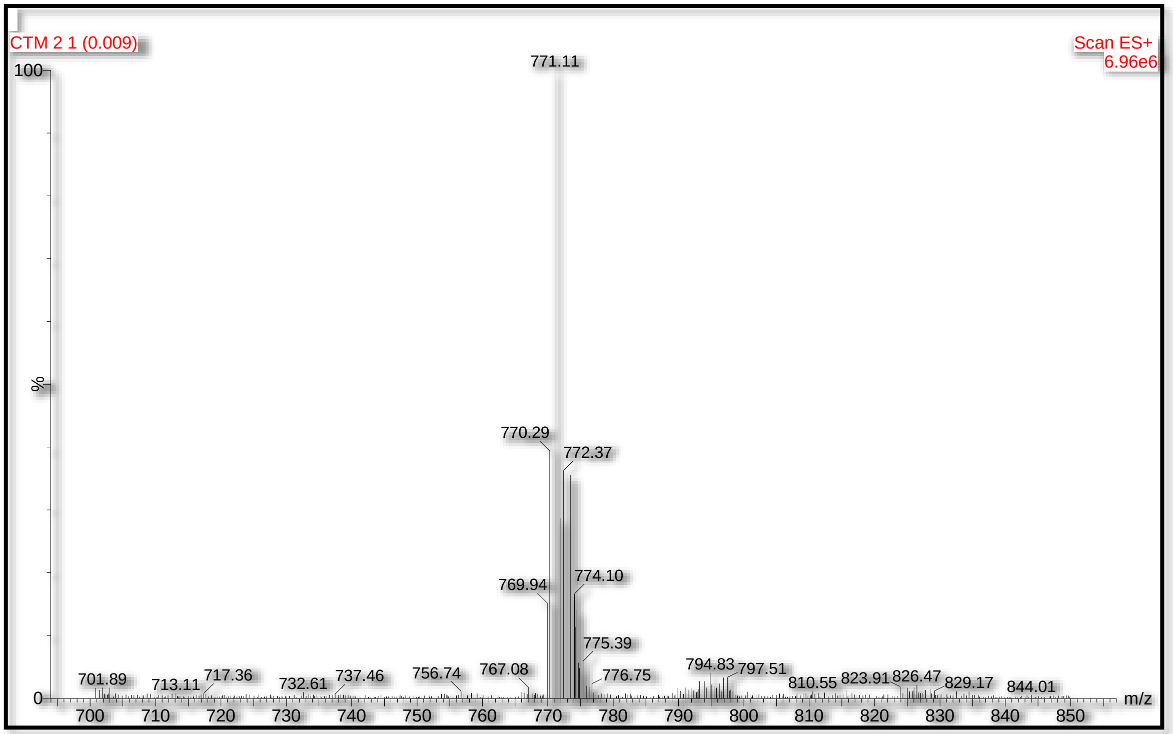

S9

1H and 13C NMR for Compound 9b

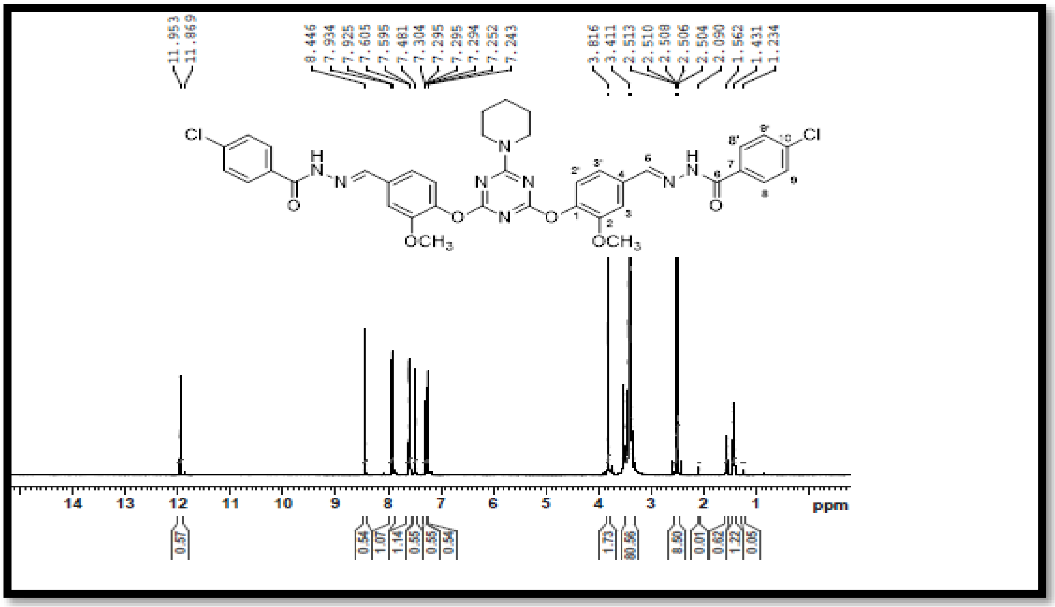

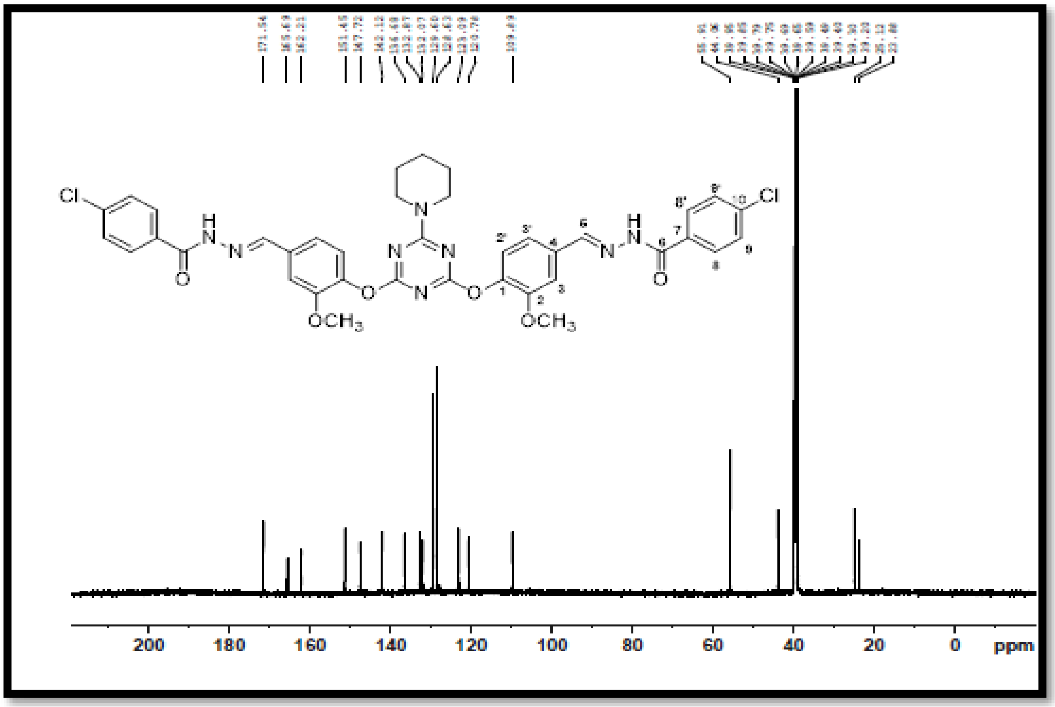

S10

UPLC-MC compound 9b

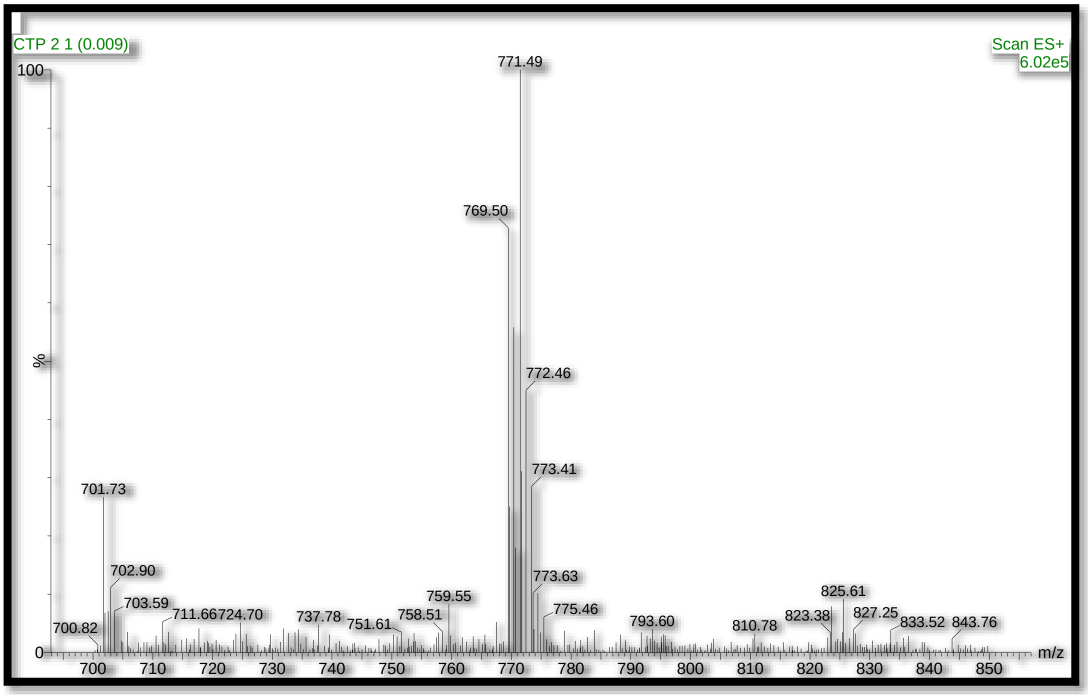

S11

^{1}H and ^{13}C NMR for Compound 9c

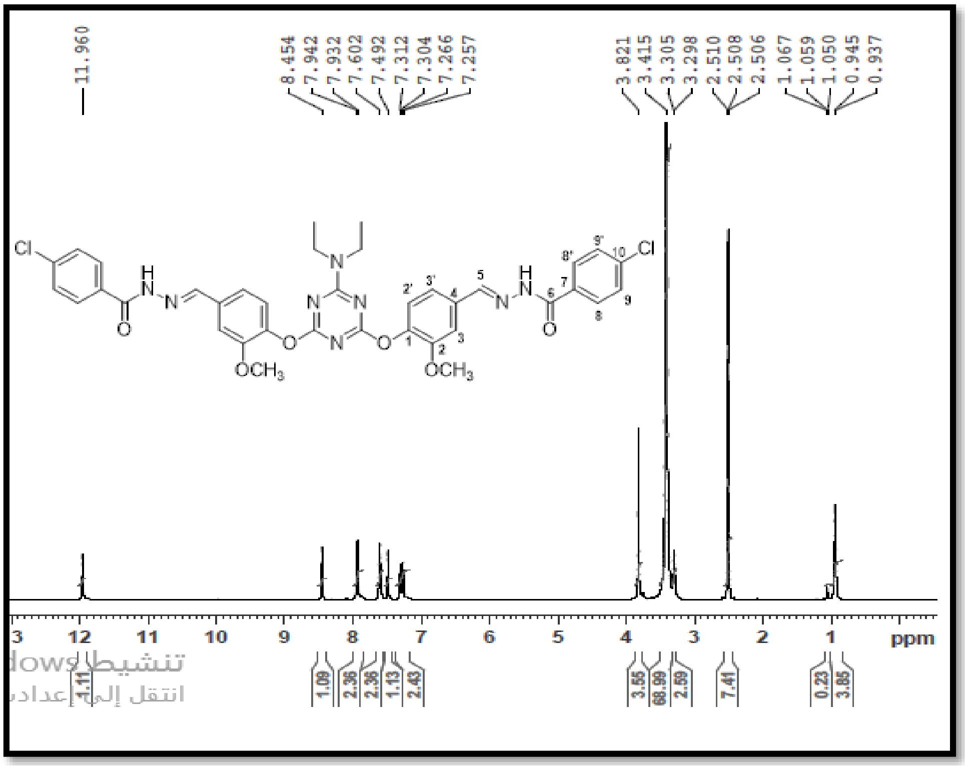

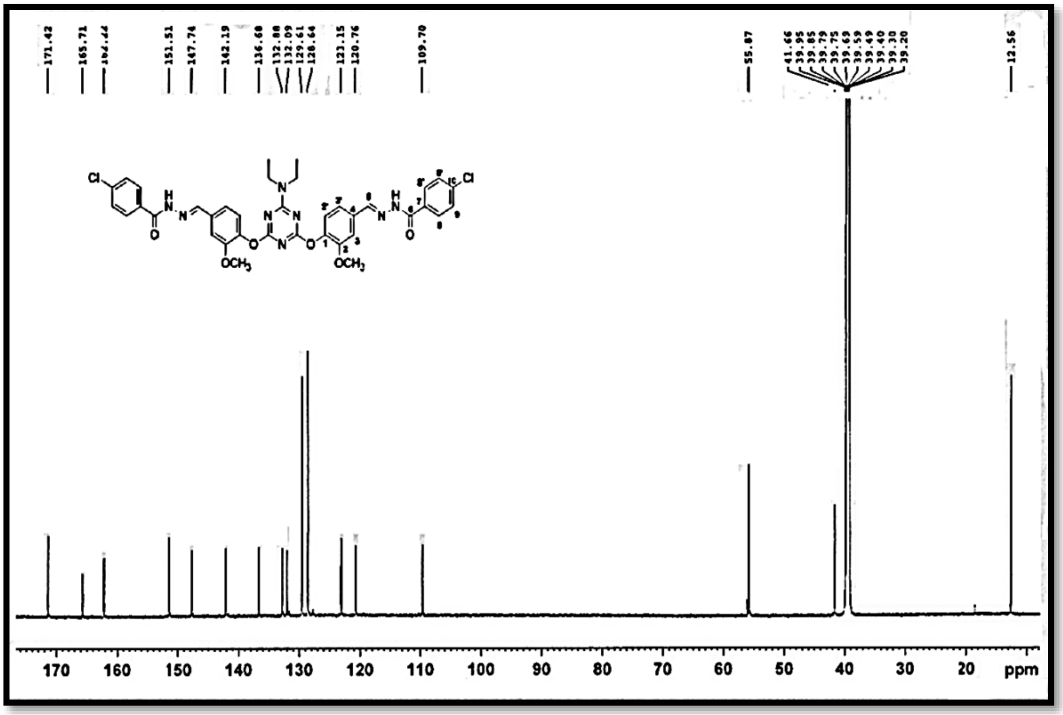

S12

UPLC-MS for compound 9c

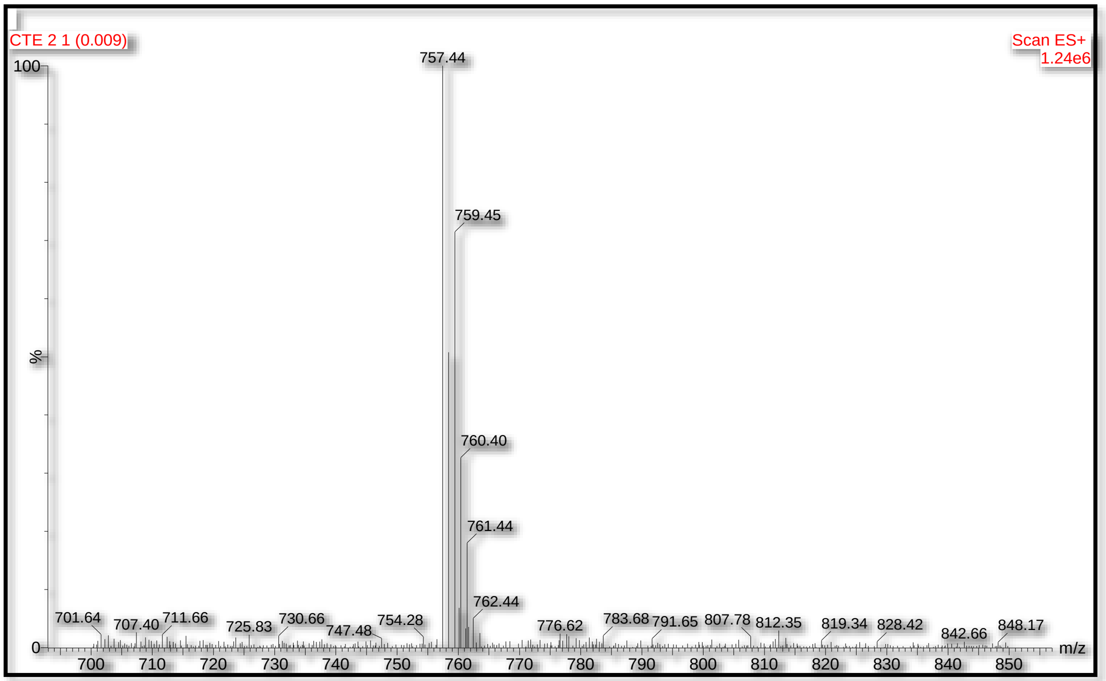

S13

1H and 13C NMR for Compound 10a

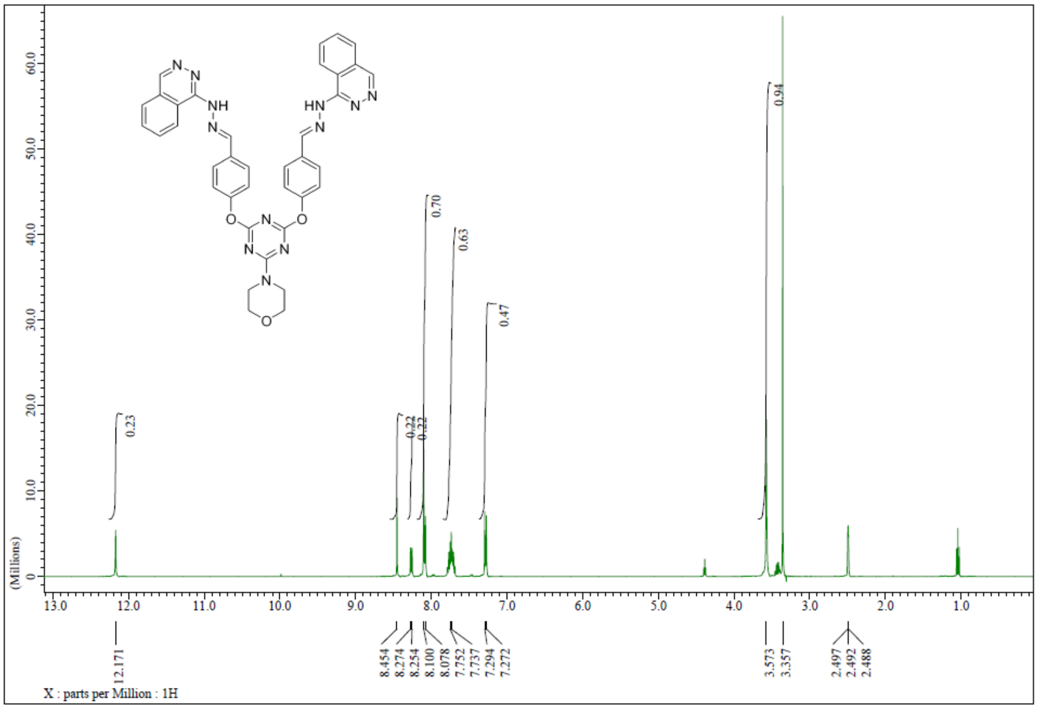

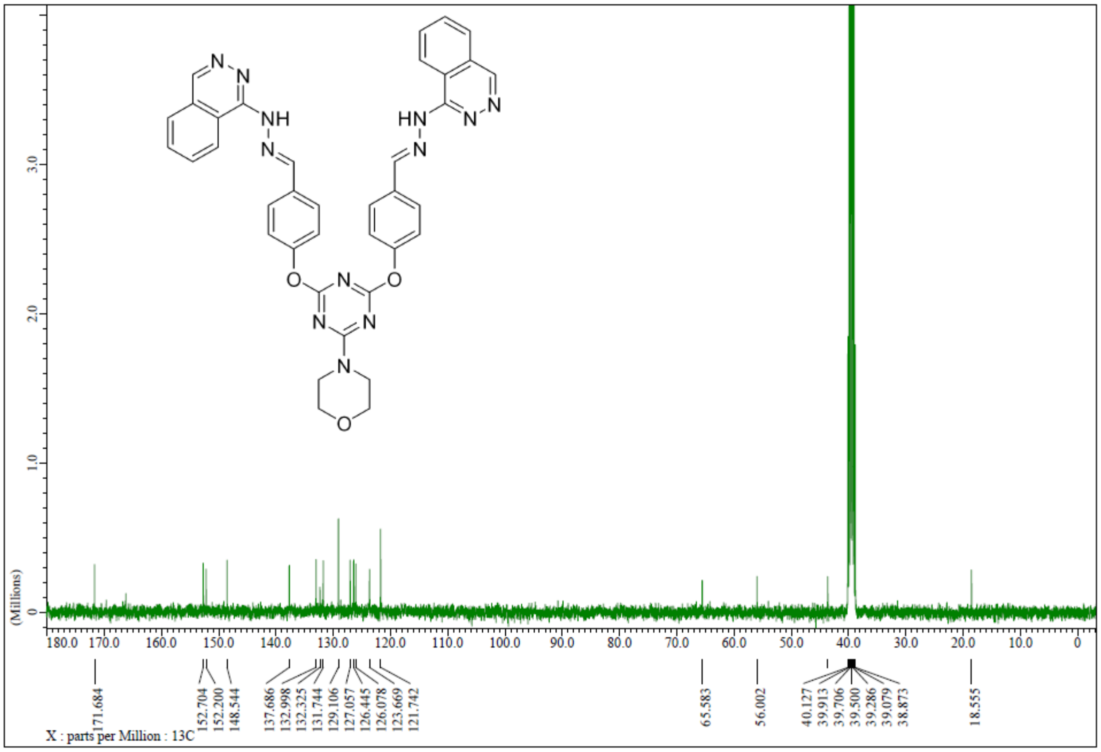

S14

1H and 13C NMR for Compound 10b

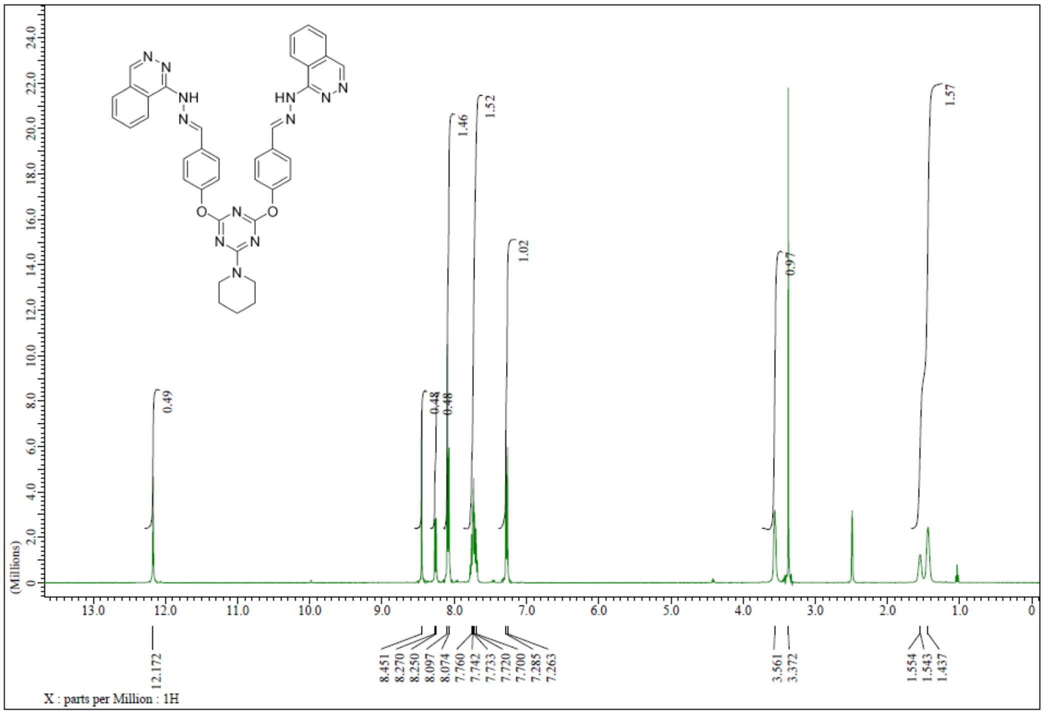

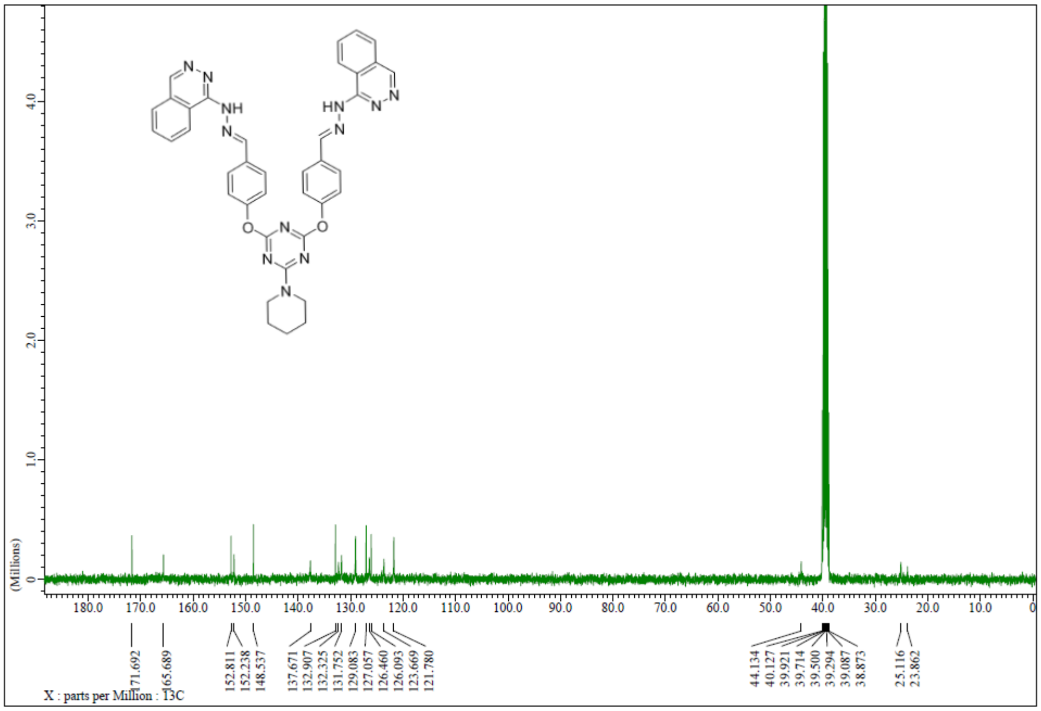

S15

UPLC-MS for compound 10b

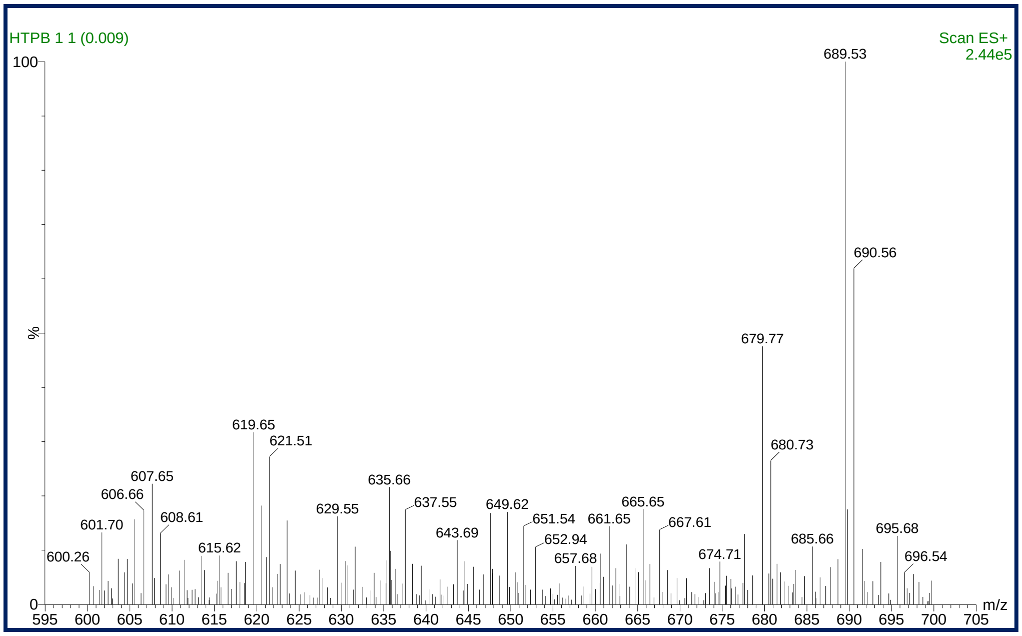

S16

1H and 13C NMR for Compound 10c

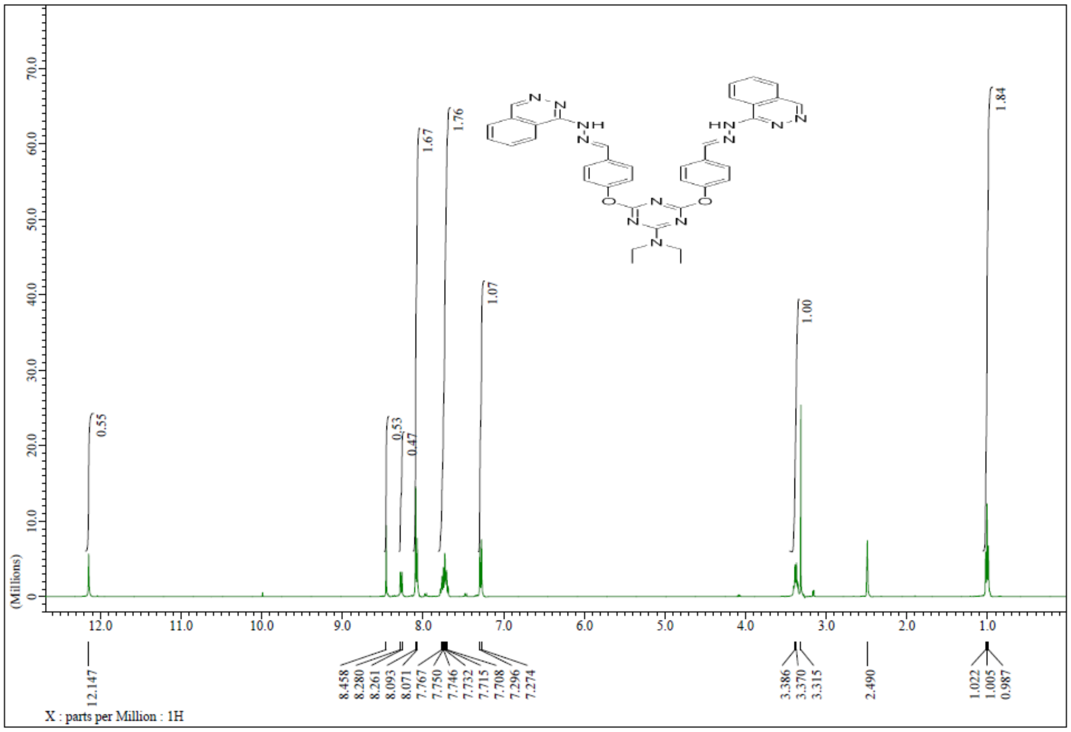

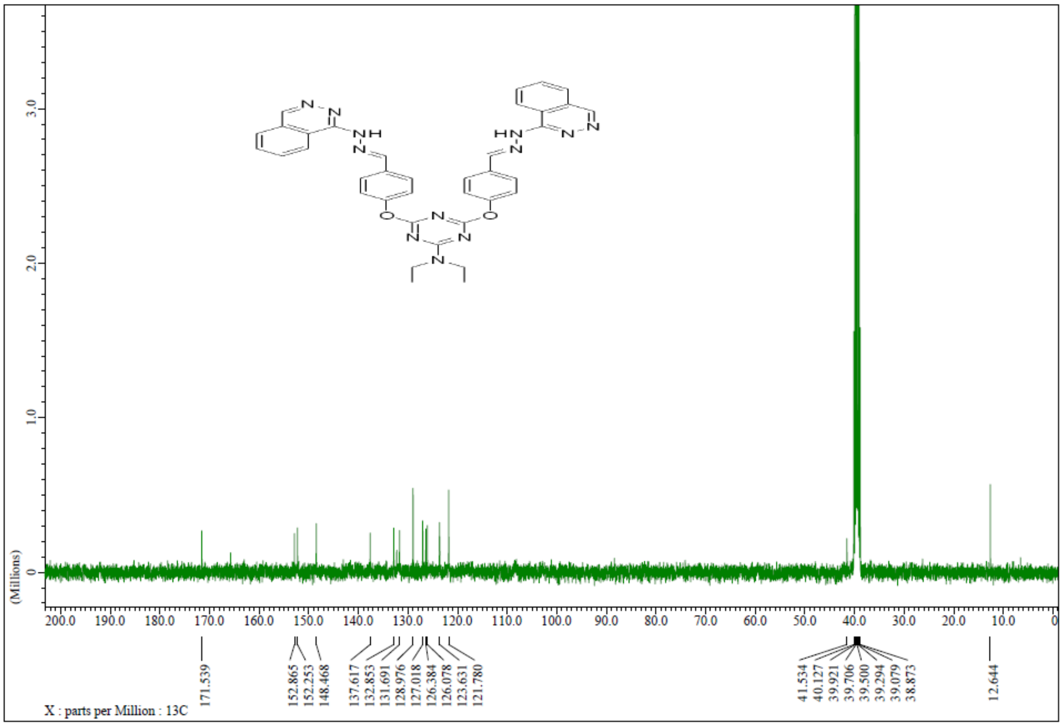

S17

UPLC-MS for compound 10c

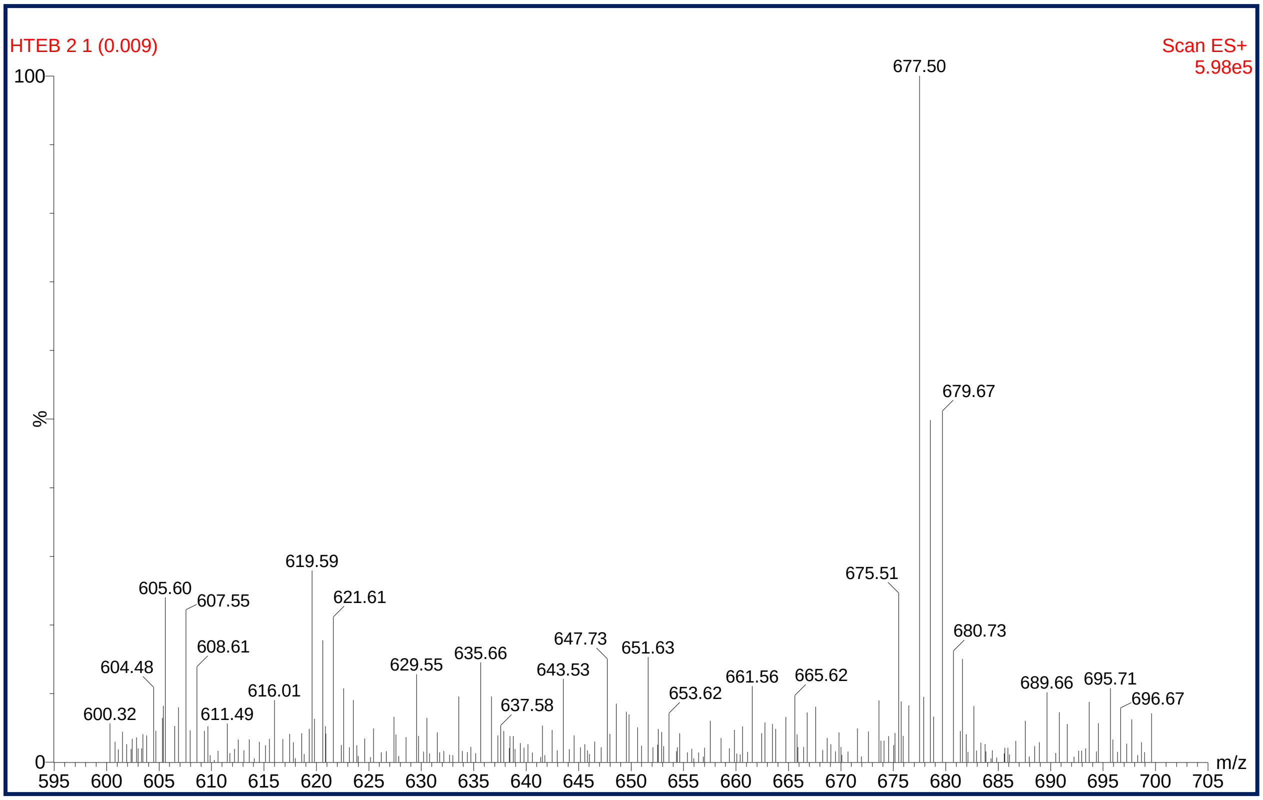

S18

1H and 13C NMR for Compound 11a

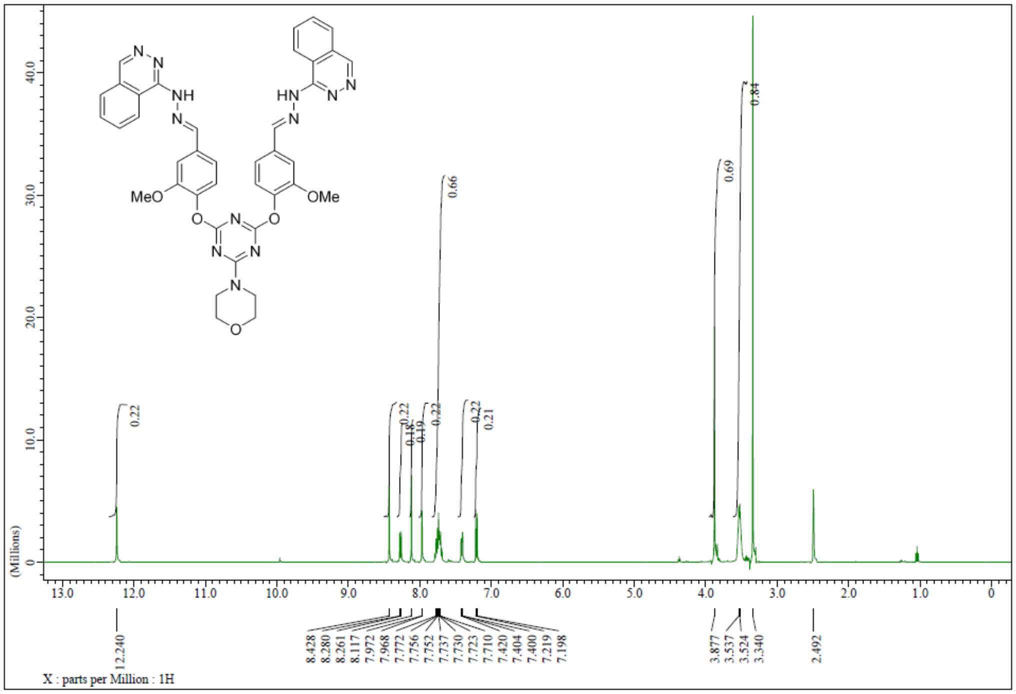

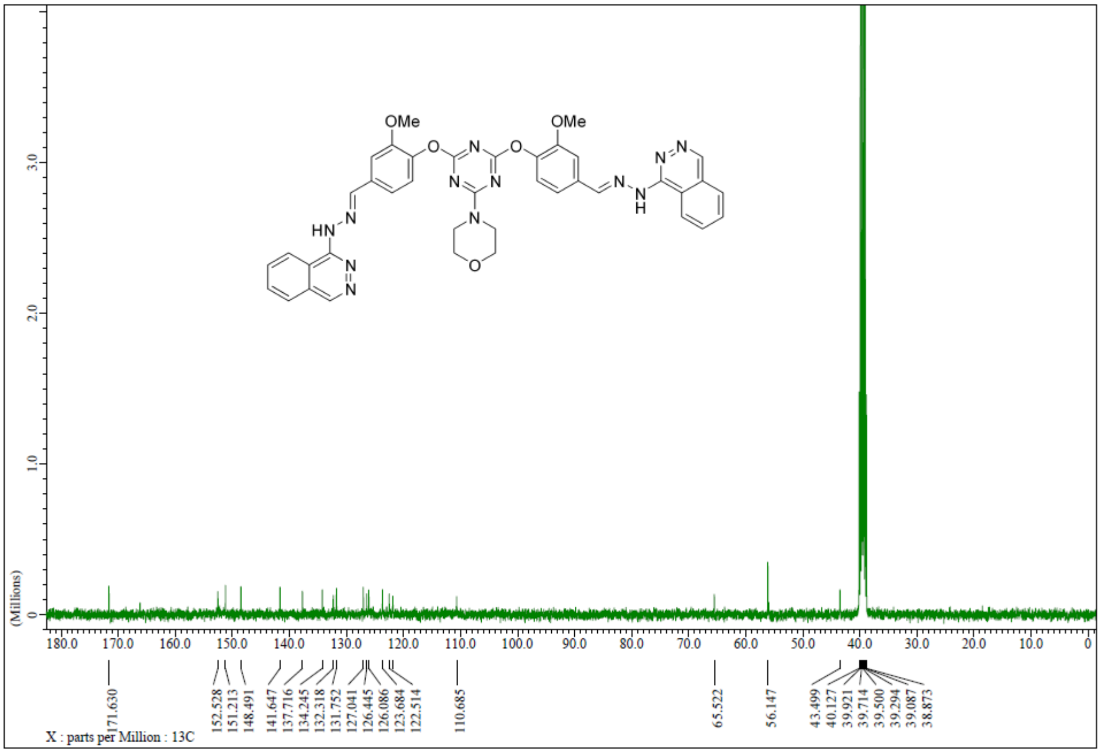

S19

1H and 13C NMR for Compound 11b

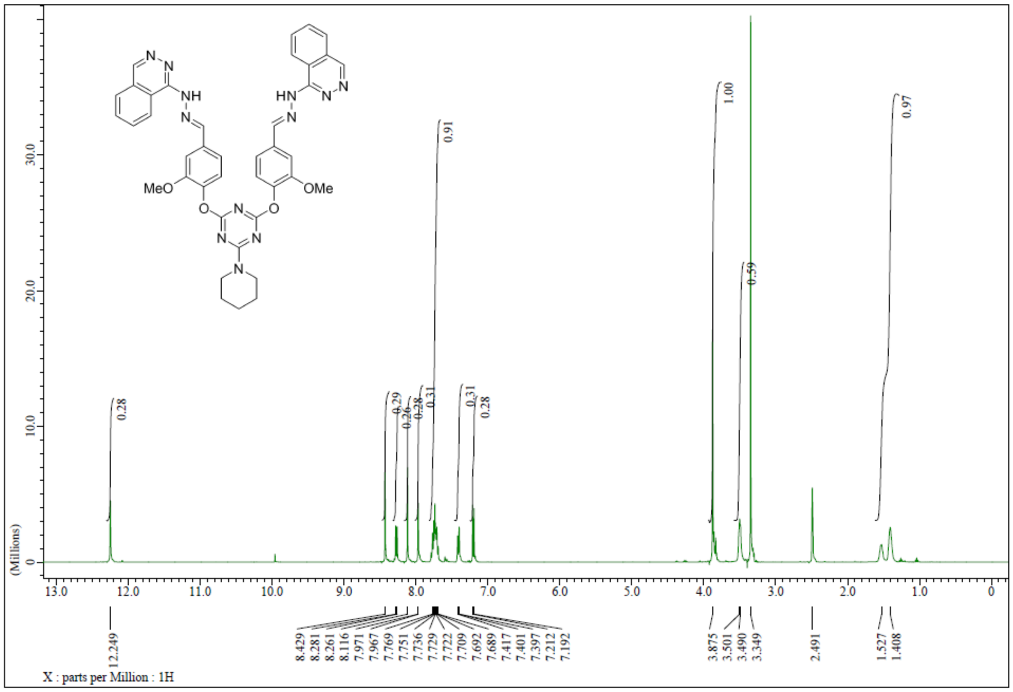

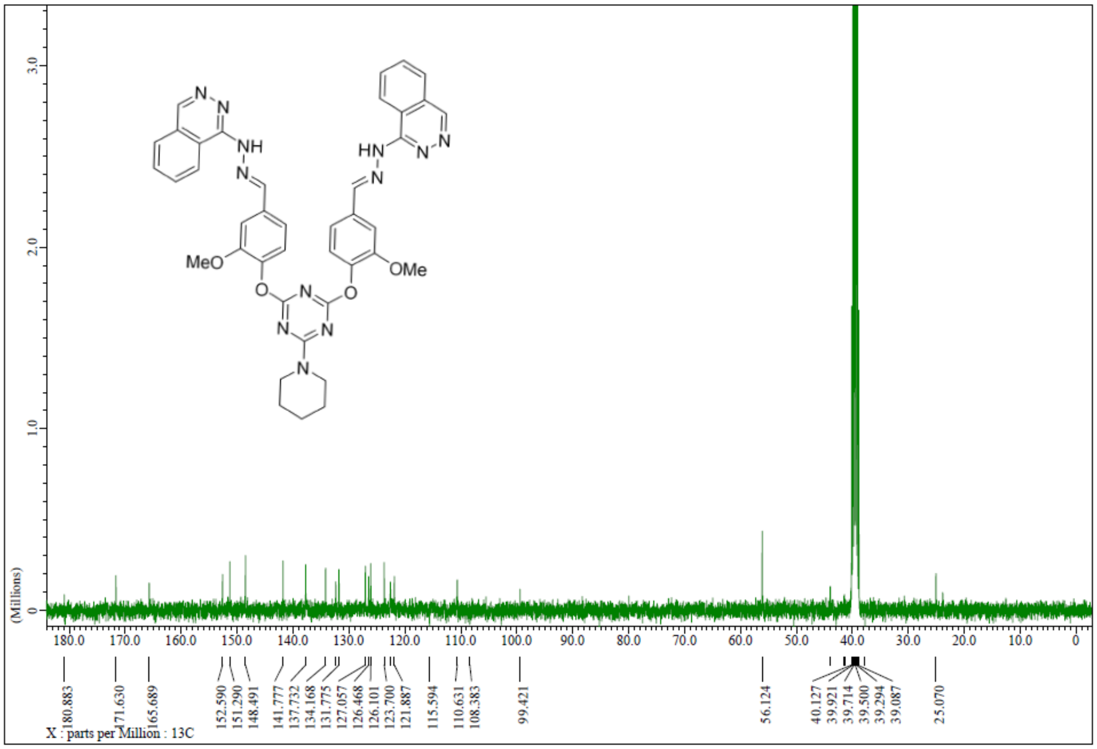

S20

UPLC-MS for compound 11b

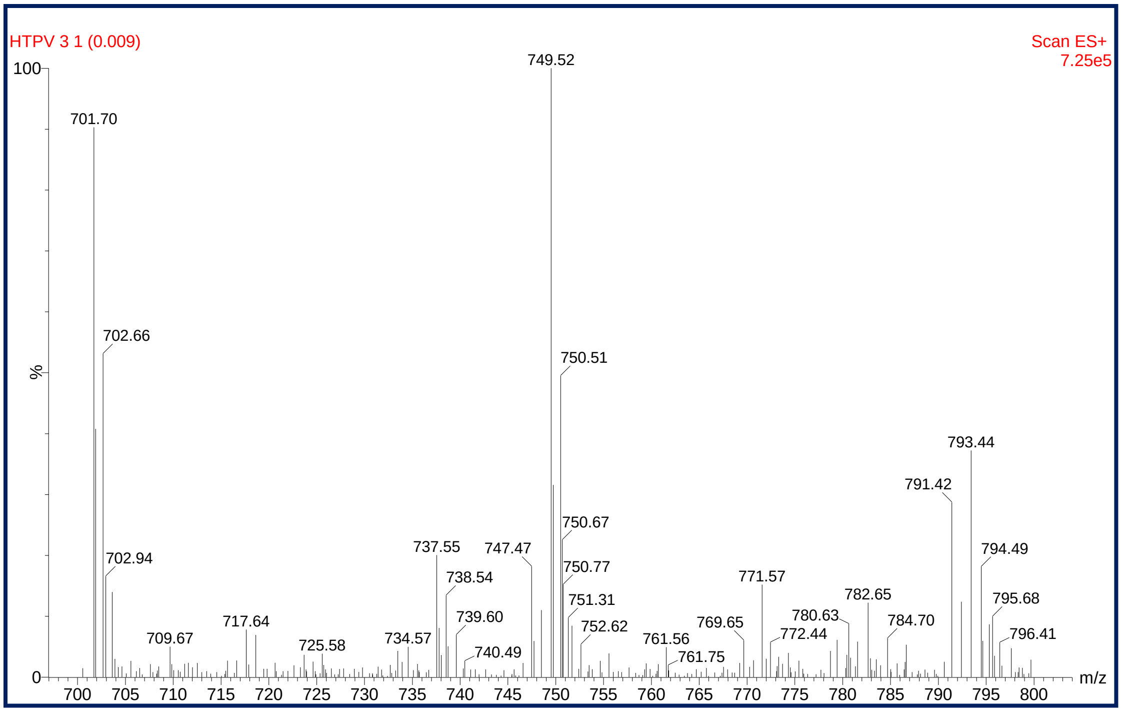

S21

1H and 13C NMR for Compound 11c

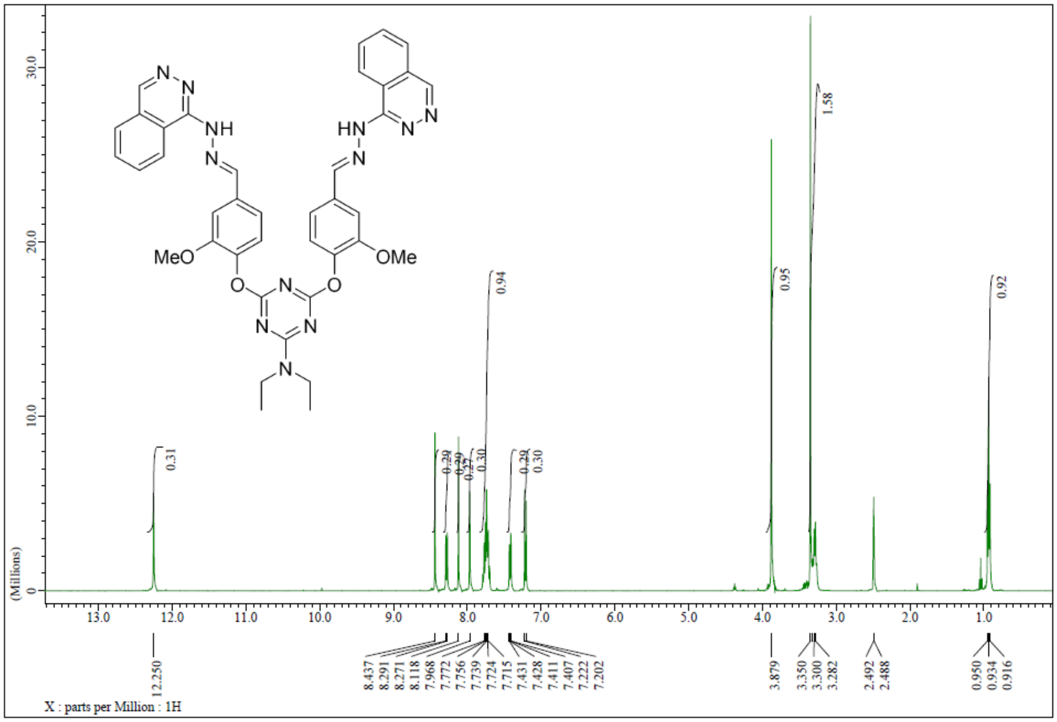

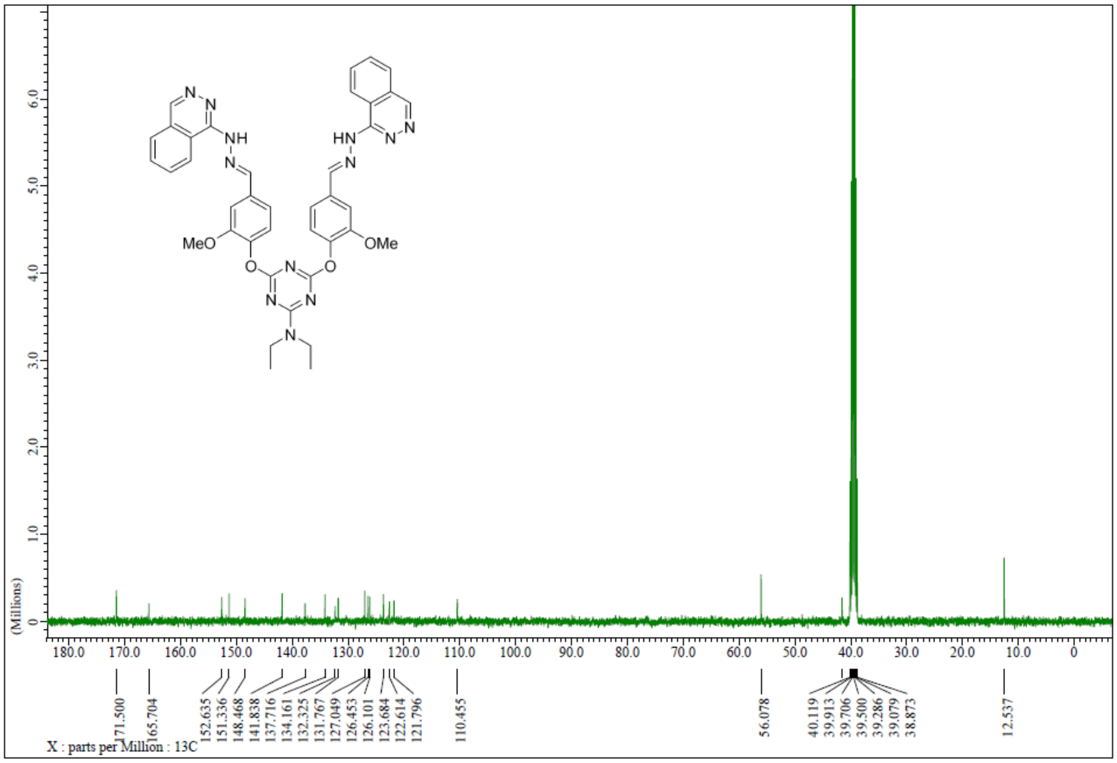

S22

UPLC-MS for compound 11c

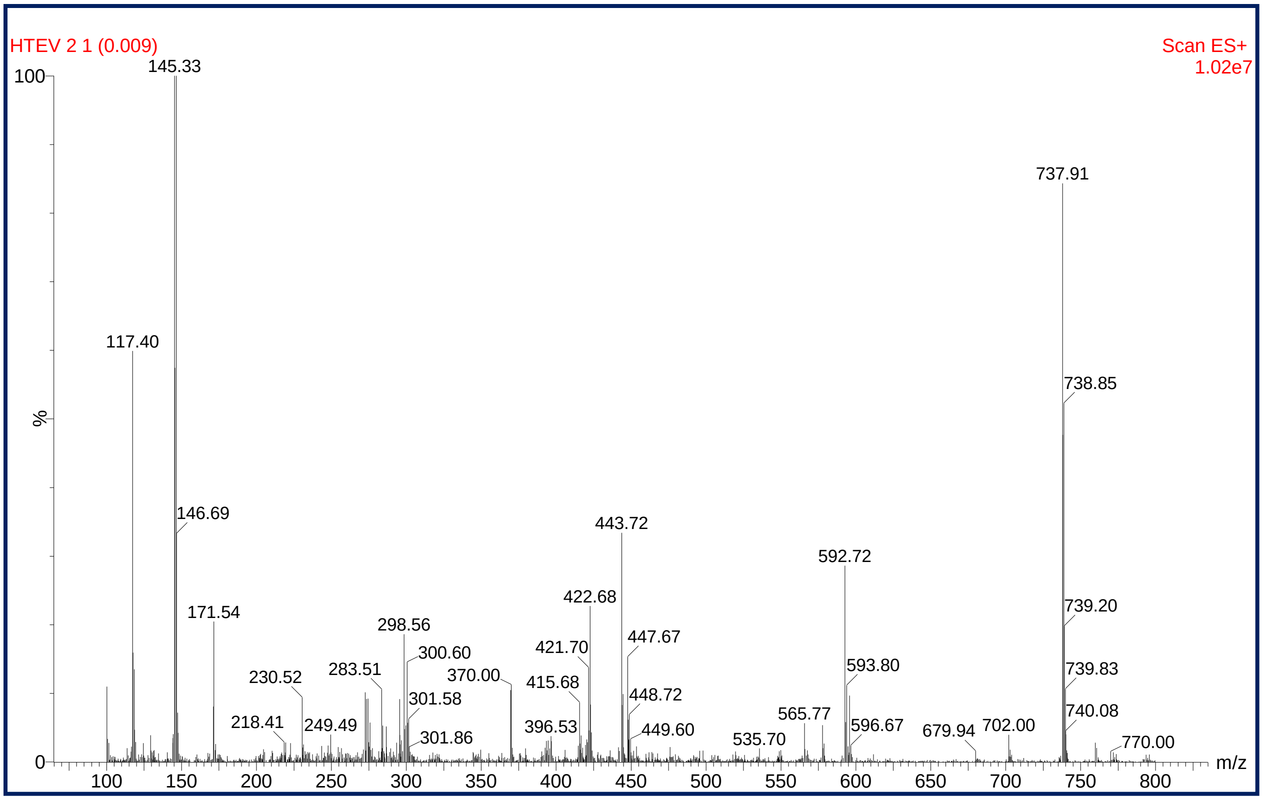

S23

1H and 13C NMR for Compound 14

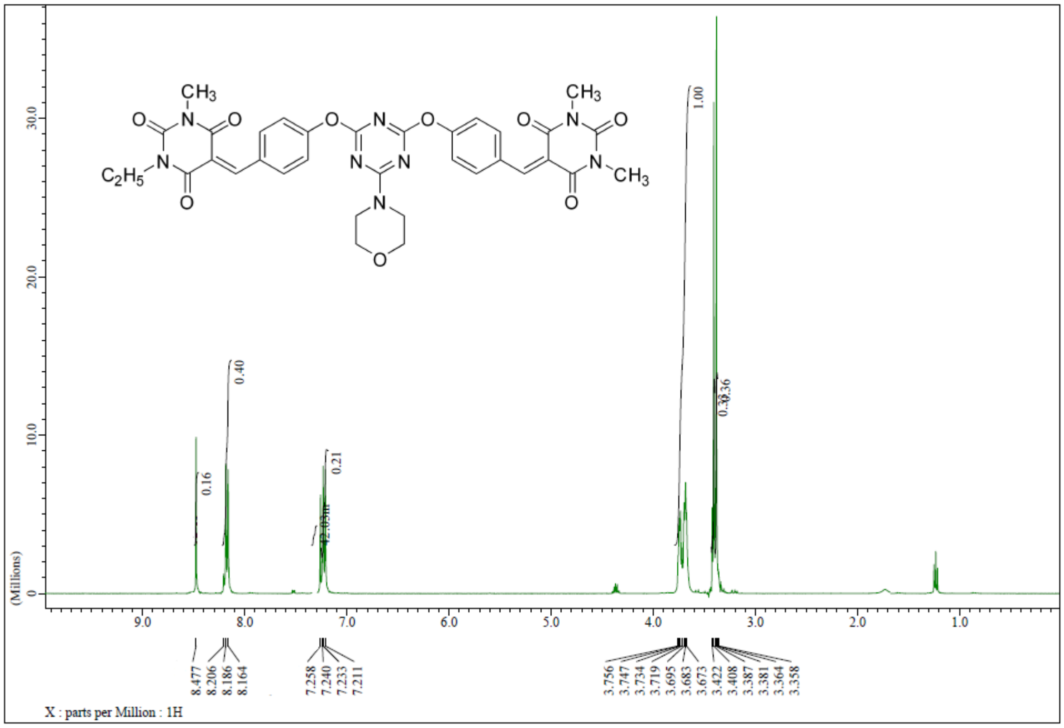

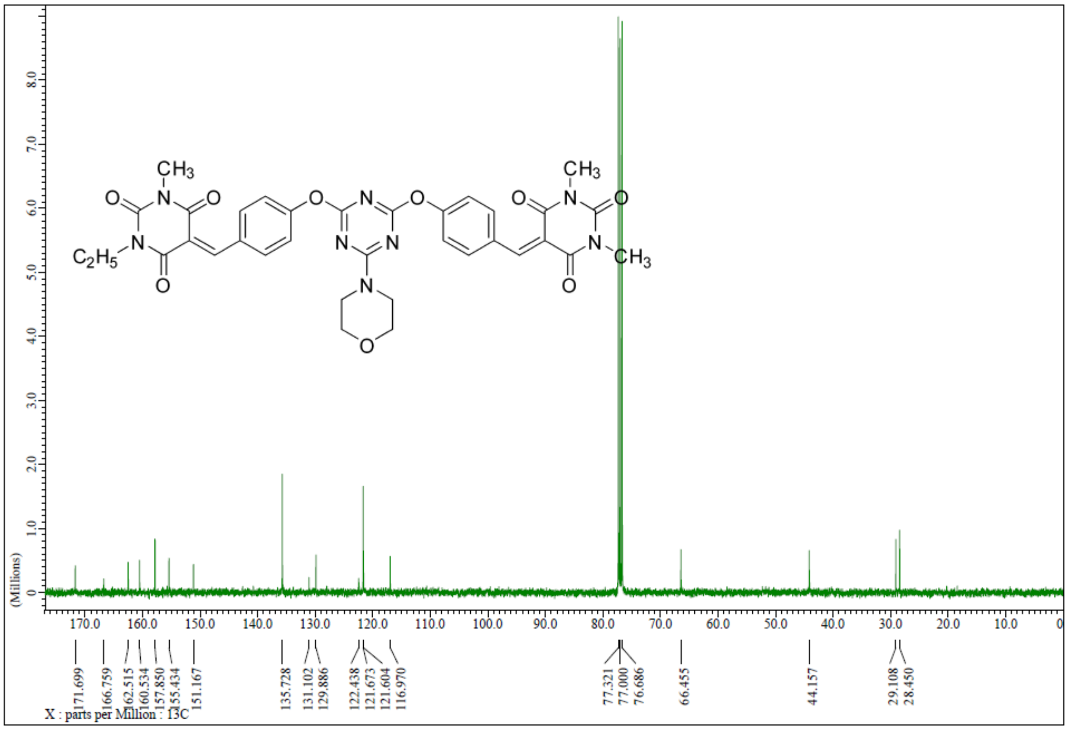

S24

1H and 13C NMR for Compound 15

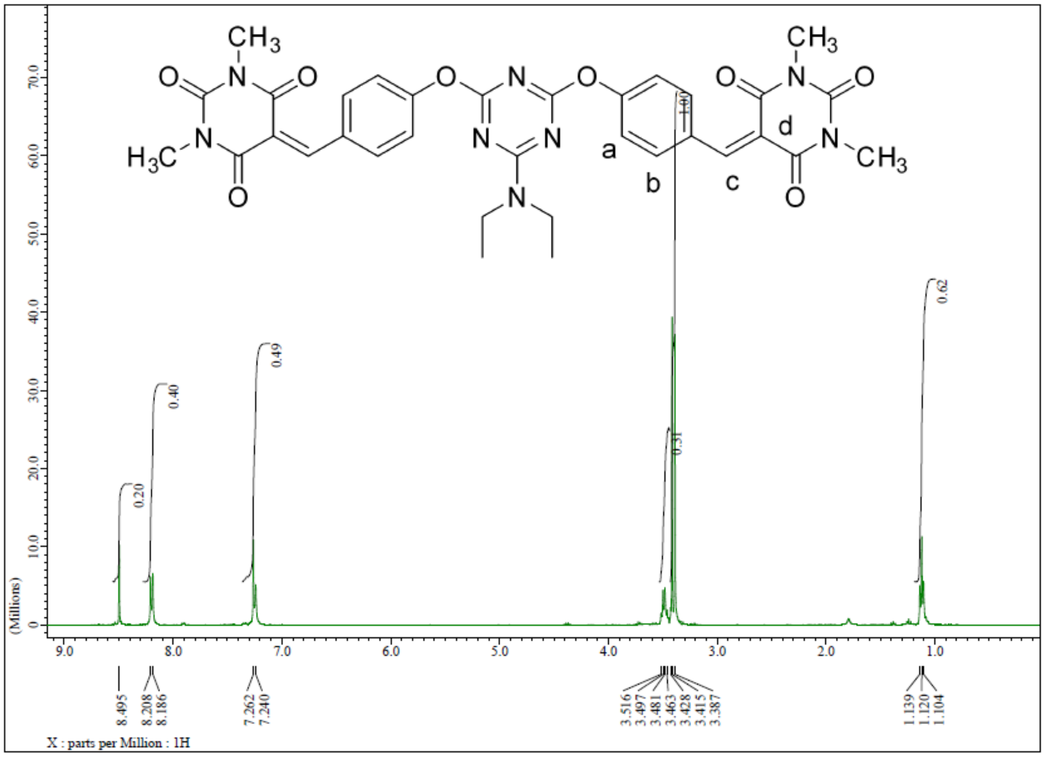

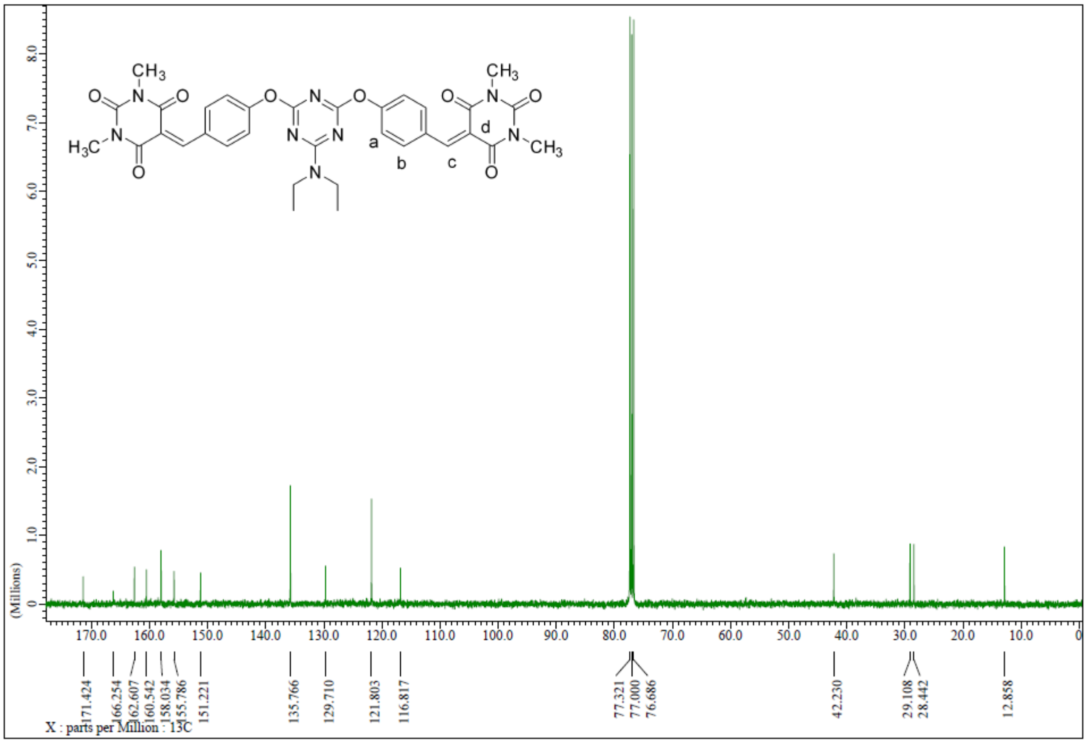

S25

1H and 13C NMR for Compound 16

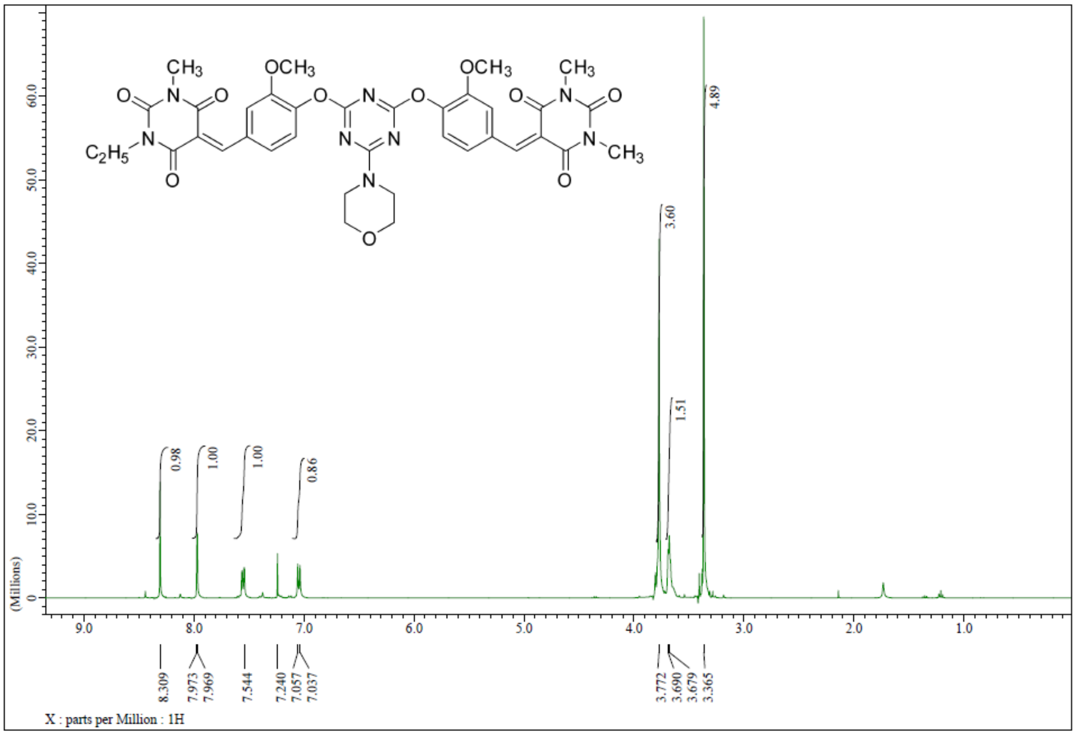

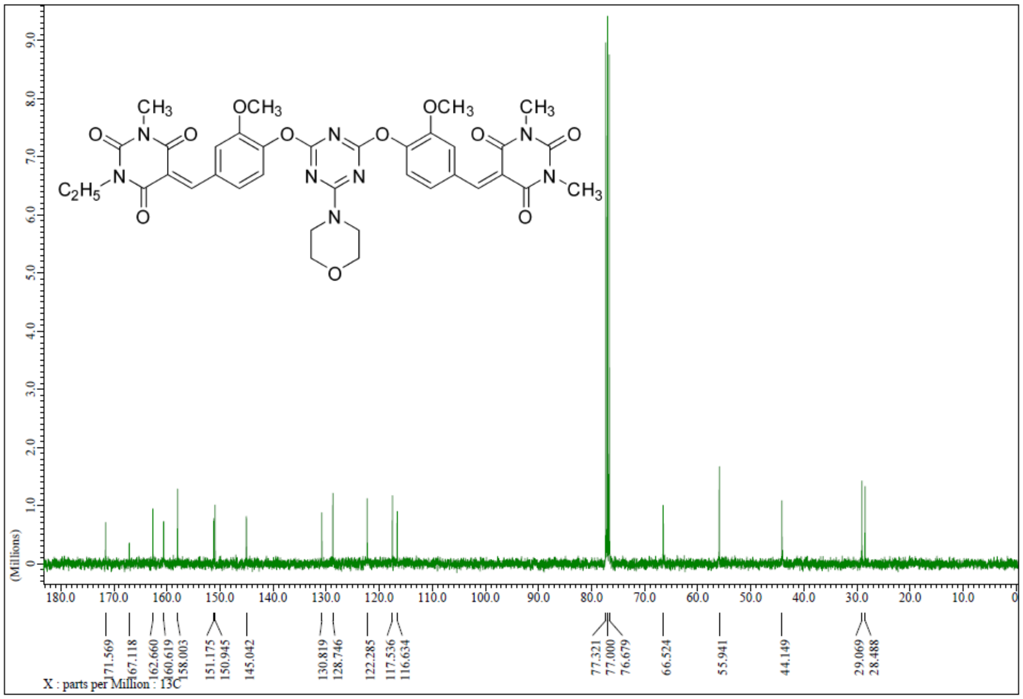

S26

1H and 13C NMR for Compound 17

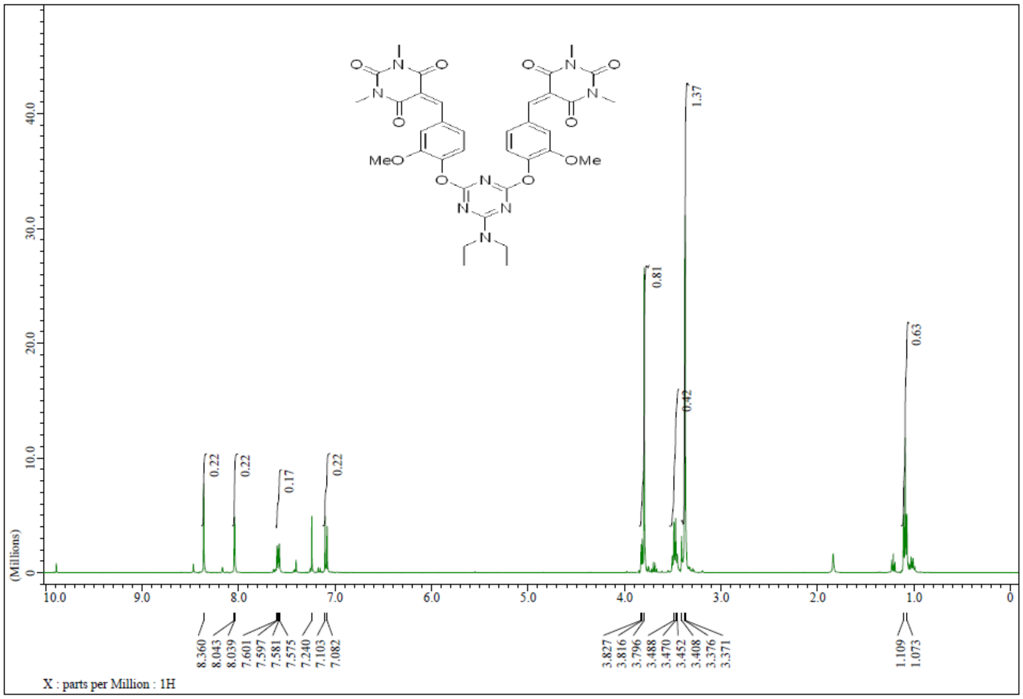

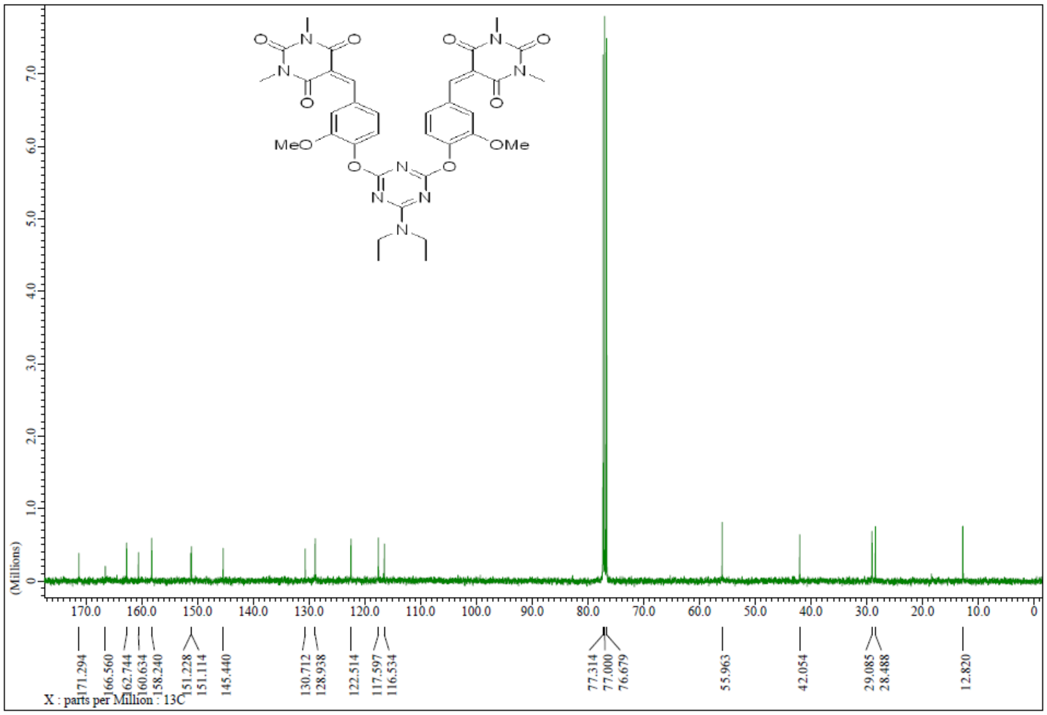

S27

1H and 13C NMR for Compound 18

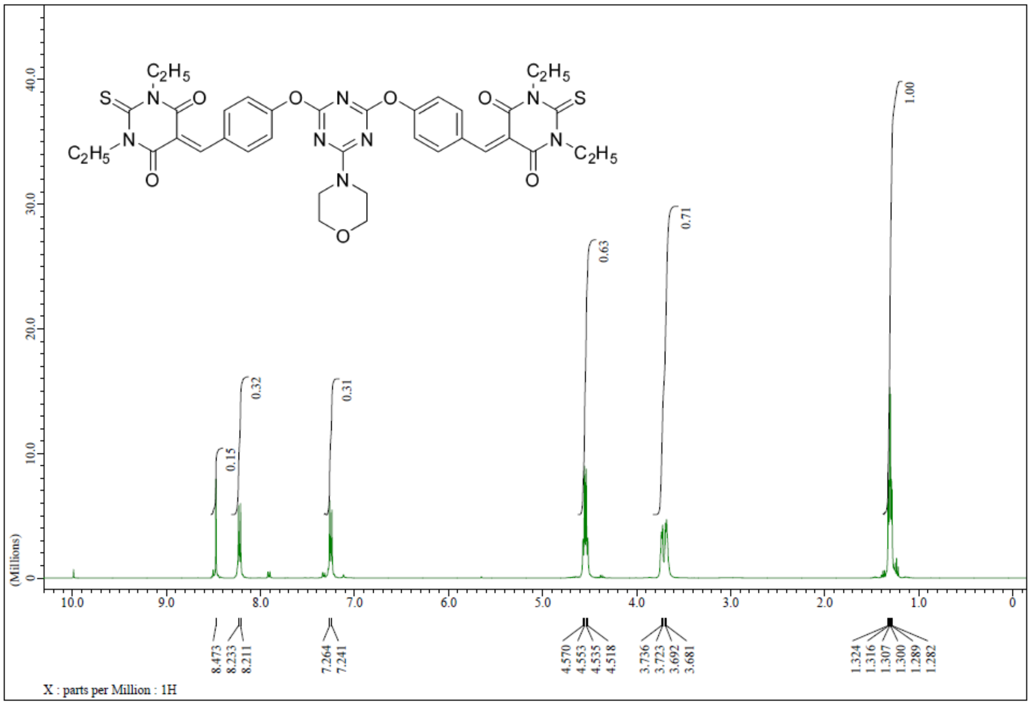

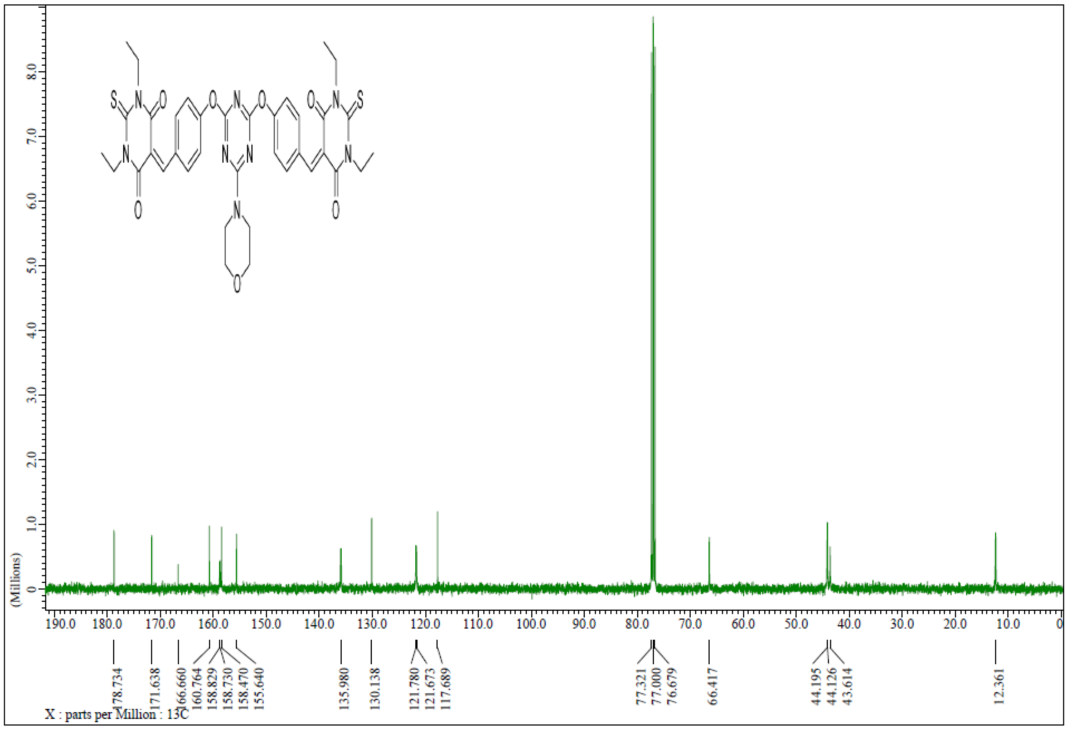

S28

1H and 13C NMR for 19

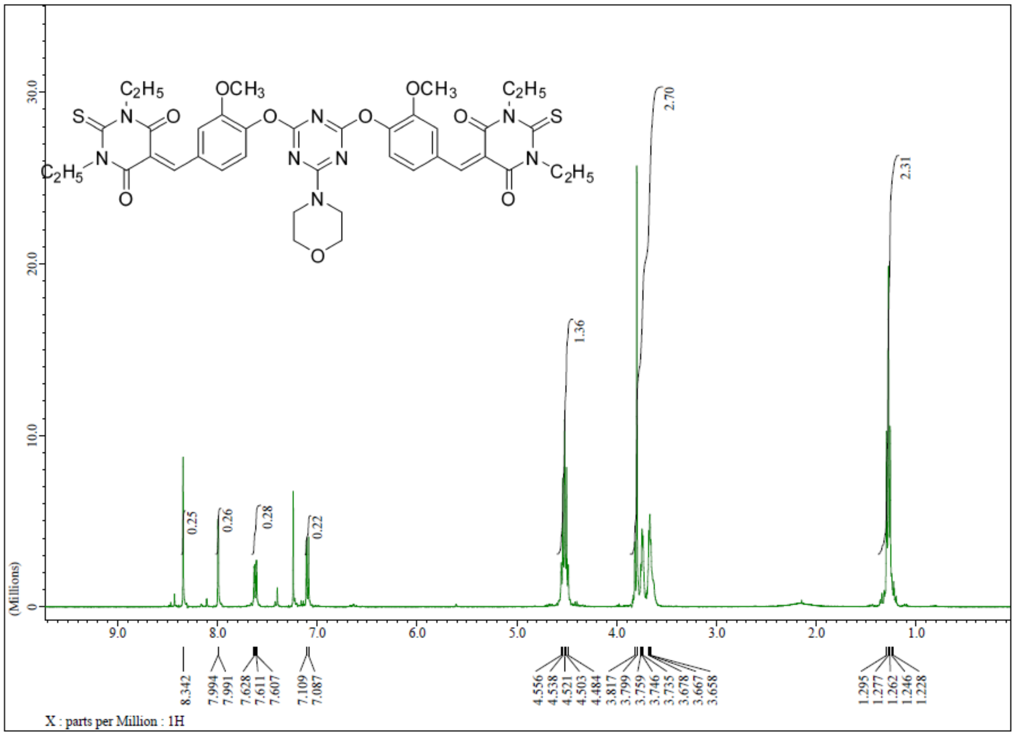

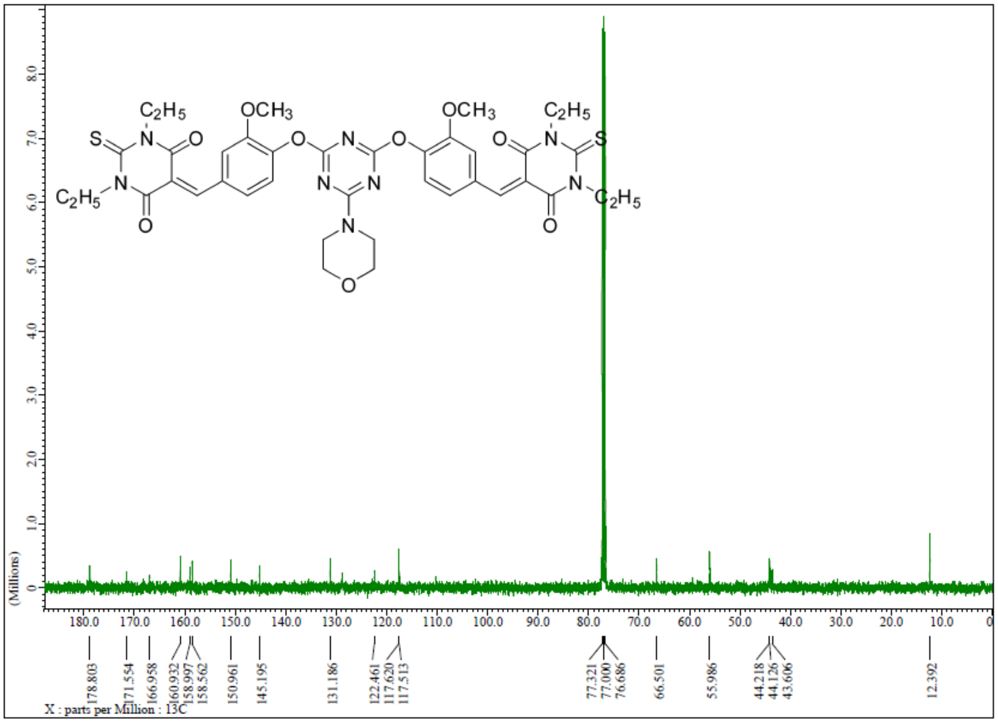

S29

1H and 13C NMR for Compound 20

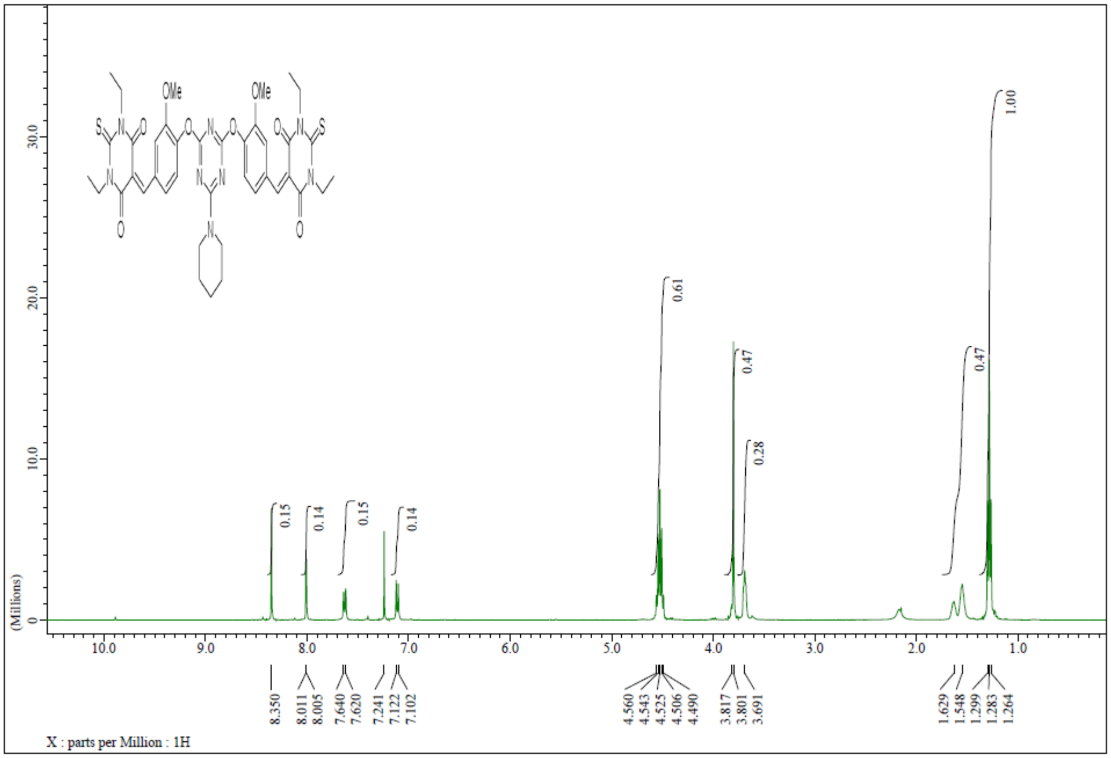

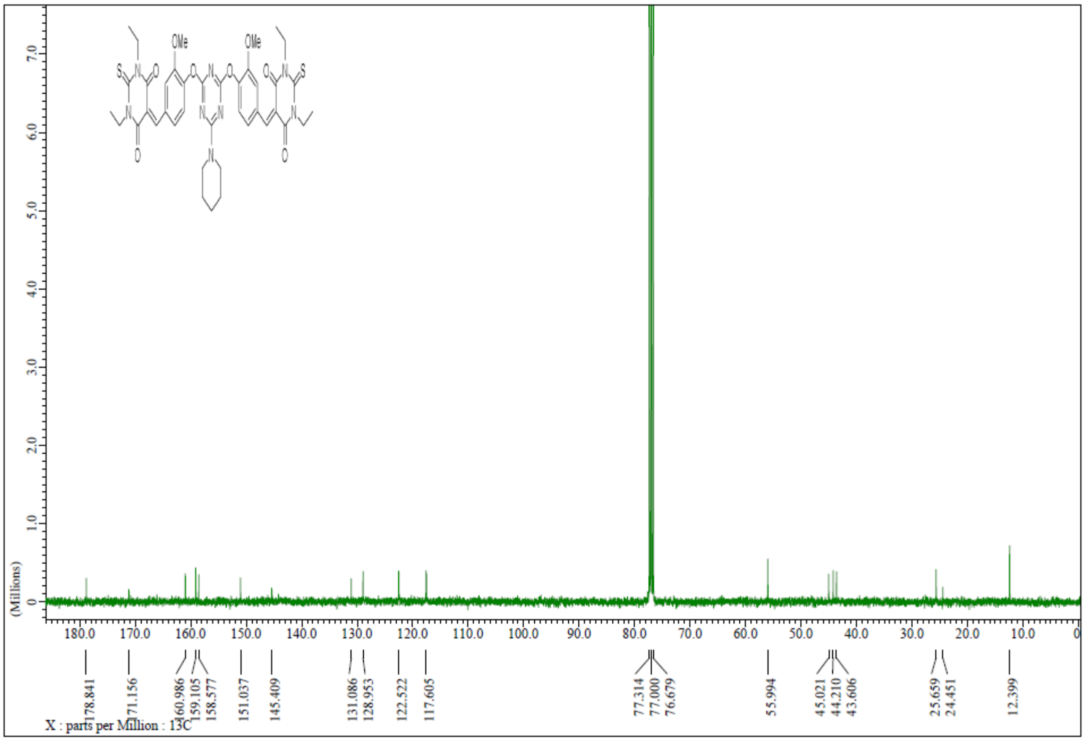

S30

1H and 13C NMR for Compound 21

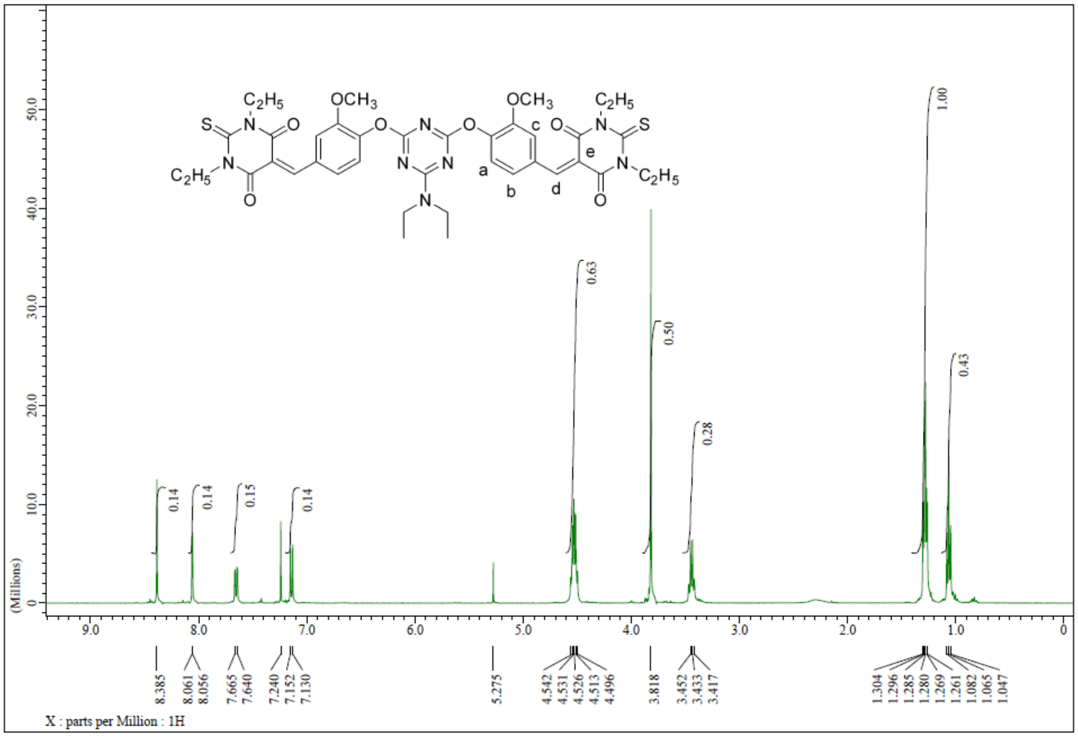

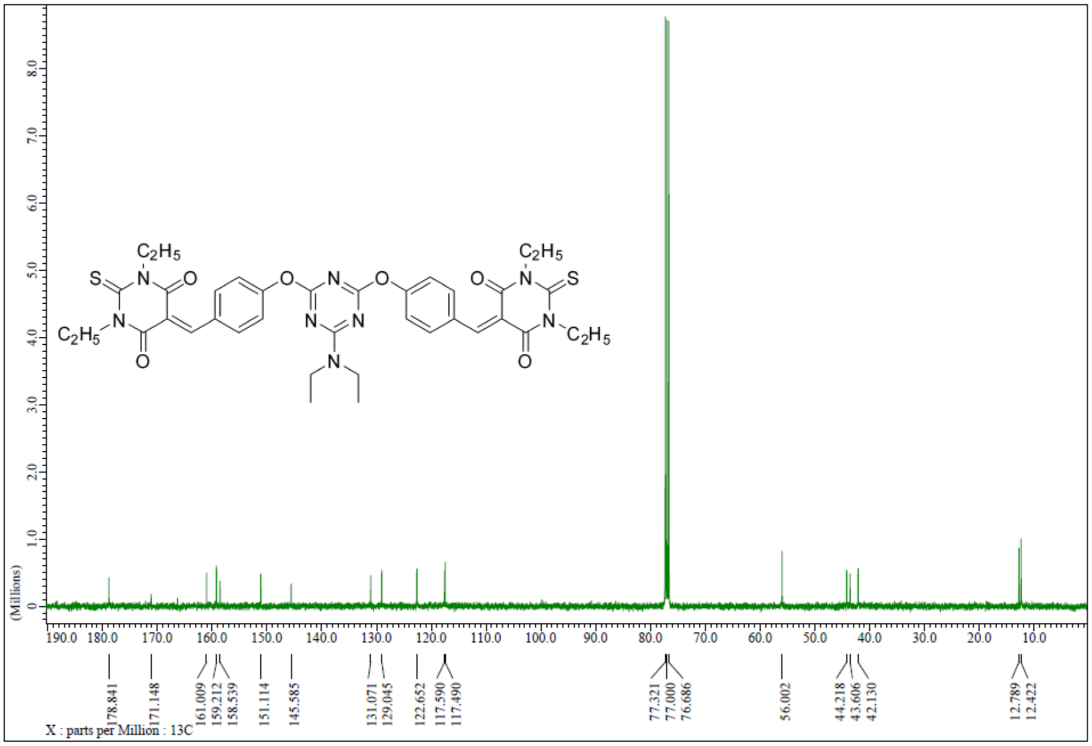

Supplement: Supplementary file 1 [file molecules-23-02976-s001.pdf]
